# p53 mutation-associated prognosis across cancer types underlines hematological malignancy as an applicable cancer type to p53-rescue therapy

**DOI:** 10.1016/j.fmre.2025.06.011

**Published:** 2025-06-30

**Authors:** Shujun Xiao, Jiaqi Wu, Kai Tan, Sujiang Zhang, Yuting Dai, Jiarui Wu, Jiale Wu, Xinle Han, Derun Zheng, Xin Wang, Huaxin Song, Min Lu

**Affiliations:** Shanghai Institute of Hematology, State Key Laboratory of Medical Genomics, National Research Center for Translational Medicine at Shanghai, Ruijin Hospital Affiliated to Shanghai Jiao Tong University School of Medicine, Shanghai 200025, China

**Keywords:** P53 mutant, P53-rescue compounds, Tumor suppressor, Cancer type selection, Target therapy

## Abstract

Tumor suppressor p53 is mutated and thereafter loses tumor-suppressive function in about 50% of cancer cases, across nearly all cancer types. Efforts to rescue mutant p53 pharmacologically have been ongoing for decades, leading to reports of approximately 71 mutant p53-rescue small-molecule compounds and > 20 clinical trials. However, no statistically significant patient benefit has been achieved, possibly due to the lack of rationalization of cancer type selection in the trials. Here, we analyzed p53 mutation-associated prognosis of overall survival, together with tumor mutation burden and concurrent cancer driver mutation with p53 mutation, across 33 cancer types in The Cancer Genome Atlas (TCGA), through which hematology malignancy was predicted as a cancer type potentially responding to p53-rescue therapy. In validation experiments, p53-null hematological malignant cell lines exhibited high sensitivity to the introduction of exogenous wild-type p53 as compared to solid-tumor cell lines. Furthermore, hematological malignant cell lines harboring structural mutant p53 are also more sensitive to the established mutant p53 rescue compound arsenic trioxide (ATO). In p53-mutant hematological malignant cells, the majority of the beneficial p53-regulating genes are upregulated while the majority of the deleterious p53-regulating genes are downregulated by ATO treatment, predicting a beneficial outcome of ATO in treating hematological malignancies in the clinic. Together, we propose a guideline for the rational selection of cancer types in the rapidly expanding p53-rescue clinical trials.

## Graphical abstract

**Figure:**
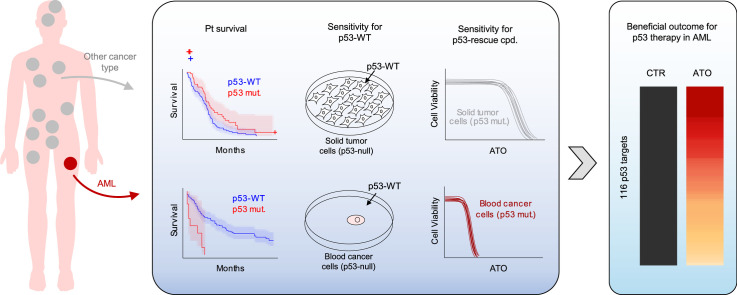

## 1 Introduction

Of the 127 genes most commonly mutated in cancer [1], approximately half encode tumor suppressors while a quarter encode oncoproteins according to OncoKB [2]. Pharmacological targeting of a mutant tumor suppressor requires challenging functional restoration rather than conventional functional inhibition. Indeed, the vast majority of the approved targeted anticancer small molecules redundantly target oncoproteins, while none target tumor suppressors, largely contributing to the situation that only 3% [3] or 2%–13% [4] of cancer patients have targeted drugs that are matched to the identified mutations in the clinic.

The major advances in the field of targeting tumor suppressors have been made on p53, the most frequently mutated protein in cancer [[5], [6], [7], [8], [9]]. To date, about 71 mutant p53-rescue compounds have been reported [8,10,11]. The majority of these 71 rescue compounds are generic rescue compounds that can rescue various p53 mutants, such as CP-31398 [12], PRIMA-1 [13], arsenic trioxide (ATO) [14], and potassium antimony (PAT) [15]. The remaining ones are p53 Y220C mutant allele-specific rescue compounds, represented by PhiKan083 [16], PC14586 [17], and KG13 [18]. Among the dozens of generic rescue compounds, ATO (and its analogues) is the only one that has scientific logic underlying rescue of unfolded p53 structural mutants—supplying “amino acid glue” that glues two or more buried p53 amino acids together and thus promote p53 folding, as seen in the most fundamental life process of the folding of polypeptides that are newly synthesized in ribosomes [14,19]. In contrast, there is no apparent scientific logic underlying the promotion of p53 folding by modification of an individual exposed cysteine, which is widely reported for the reported mutant p53 rescue compounds [20], because such modification does not supply “amino acid glue”. Consequently, among the evaluable mutant p53 rescue compounds, ATO and its analogues such as PAT and AcGlcAs [14,15,21] stand out due to their reproducible effectiveness: in 10 widely used p53 assays side-by-side comparing accessible mutant p53 rescue compounds, none of the them is effective in rescuing mutant p53 in any of these 10 assays, except for ATO and its analogs [22].

Among the reported ∼71 p53-rescue compounds, APR-246, ATO, PAT, COTI-2, Kevetrin, PEITC, and PC14586 have progressed into about 20 registered clinical trials, covering over 1000 cancer patients [[5], [6], [7], [8], [9]]. In the first-in-human clinical trial of p53-rescue therapy in 2012, APR-246 demonstrated a partial response (PR) rate of 2/22 [23], and currently, thirteen APR-246 clinical trials are registered in ClinicalTrials.gov. Another milestone is the first-in-human mutant p53 reactivation in 2023, where specific and global upregulation of the set of p53 target genes was observed in an ATO-treated p53-mutant non-APL leukemia patient [24]. Among the 6 generic rescue compounds under clinical trials, ATO (and its analogue PAT) stands out as they meet the four clinical norms for advancing a laboratory-identified targeted small-molecule compound into clinical trials [19]: availability of an explicable structural model to ensure its targeting mode [14,15], compatibility of SAR with proposed structural model to ensure correctness of the structural model [15,21], target (namely mutant p53) specificity in cells to ensure it can bind its target in the human body [24], and a complete list of applicable p53 mutant alleles to enable precise patient recruitment [15,24]. In targeted oncology, these four norms are rigorously followed by the ∼85 approved targeted drugs, the ∼495 targeted small molecules that enter clinical trials, and even the wild-type (WT) p53 function-boosting compounds (namely, MDM2 inhibitors). However, these four norms are poorly adhered to when clinically translating the numerous generic mutant p53 rescue compounds, except for ATO and its analogue. Since 2023, ATO has become the most intensively trialed mutant p53 rescue compound in the clinics [7].

Despite the large number of p53-rescue clinical trials, no statistically significant patient benefit has been achieved to date. This disappointment may be attributed to the unique property of p53 mutations in cancers. p53 is a unique protein in the proteome, featured by thousands of diverse mutations across the protein sequence and, in addition, across nearly all cancer types. These features pose two challenges in the design of p53-rescue clinical trials — the rationale selection of p53 mutations and cancer types. We recently conducted a quantitative assessment of the potencies of ATO in rescuing 800 most common cancer-associated p53 missense mutations, through which we identified 390 p53 mutations that can be effectively rescued by ATO [24]. Based on these findings, we propose a guideline for precise selection of p53 mutations in ATO clinical trials, namely exclusively recruiting cancer patients harboring one of these 390 rescuable mutations (www.rescuep53.net). Yet, to the best of our knowledge, the selection of cancer types has not been rationalized in any p53-rescue trials, despite the established knowledge that the tumor-suppressive function of p53 is highly context-dependent. The lessons learned from the high treatment efficacy of BRAF inhibitors in V600E-mutant melanoma, but not colorectal cancers harboring the same mutant [25], highlight the importance of selecting an appropriate cancer type when designing clinical trials for a targeted drug.

In this study, we analyzed p53 mutation-associated prognosis factors across 33 cancer types in TCGA, through which we predicted hematological malignancy as ideal cancer types potentially responsive to p53-rescue therapy. In further validation experiments, we confirmed that hematological malignant cell lines showed the greatest sensitivity to not only WT p53, but also ATO-rescued mutant p53. Our study addresses a critical aspect of p53-rescue therapy—rational cancer type selection—and thus potentially accelerates the clinical approval of p53 rescue drugs.

## 2 Methods

### 2.1 Analysis of high-frequency mutated genes in pan-cancer

First, the 40 most frequently mutated genes across pan-cancer were identified from the cBioPortal (https://www.cbioportal.org/), and classified into four types using OncoKB (https://www.oncokb.org/cancer-genes): oncogenes, tumor suppressor genes (TSG), genes functioning as both oncogenes and TSGs (Both) and genes that are neither (No). Subsequently, we retrieved these 40 genes across 31 cancer types from cBioPortal and employed R to generate the bubble chart to visualize the mutation frequencies of these genes.

### 2.2 TCGA pan-cancer atlas data analysis

RNA-seq gene expression, clinical characteristics, and mutation data from the TCGA Pan-Cancer Atlas, comprising 9875 primary tumor samples across 33 tumor types, were obtained from UCSC Xena (https://xenabrowser.net/datapages/). Gene expression levels were quantified using FPKM to ensure consistency across samples. Somatic p53 mutations were identified based on mutation-calling results from four pipelines: MuSE, MuTect, SomaticSniper, and VarScan. Survival curves for each TCGA cohort were generated using the Kaplan-Meier method, and statistical significance of difference was assessed using the log-rank test. The median values for gene expression were evaluated in relation to patients’ overall survival, progression-free interval and disease-specific survival within each TCGA cohort, utilizing the survminer R package (https://CRAN.R-project.org/package=survminer). Hazard ratios (HR) for univariate analysis were calculated using the Cox proportional hazards regression model through R package survival (https://CRAN.R-project.org/package=survival). All statistical analyses were performed using R 4.2.1. The *P*-values were two-tailed, and *P*-values below 0.05 were considered statistically significant.

### 2.3 Tumor mutational burden analysis

Somatic mutations in the TCGA cohort were identified using four pipelines: MuSE, MuTect, SomaticSniper, and VarScan. We use 38 Mb as the estimate of the exome size (PMID: 28420421, 30505710). Tumor mutational burden (TMB) was calculated as the observed number of mutations per Mb.

### 2.4 Cell growth assay for p53-null cell lines upon introduction of wild-type p53

For wild-type p53 and vector retroviral construction, 2 × 10^6^ HEK293T cells were seeded on a 10 cm dish for 16 h, followed by co-transfection with two helper plasmids (6 mg VSV-G and 9 mg gag-pol) and 16 mg of wild-type p53 or vector plasmids. Transfection was performed using Hilymax transfection reagents (H357, Dojindo) according to the manufacturer’s instructions and the medium was replaced after 4 h Retrovirus containing medium was collected at 48 h post-transfection. For stable cell line construction, 2 × 10^5^ p53-null cells were infected with 2 ml retrovirus containing medium by spinning in a 6-well plate at 2000 rpm for 2 h in the presence of 8 mg/mL polybrene (AL-118, Sigma). Six hours later, 4 ml fresh medium was added to the original virus containing medium. Cells were counted under a microscope on days 1, 3, and 6 post-infection. For each cell type, three random fields were selected for counting.

### 2.5 Chip-seq analysis

Raw Chip-seq data were seen in the GEO database (GSE86164, GSE113338, GSE46991 and GSE46992) and analyzed. After cutting adaptor and filtering low-quality sequenced reads via fastQC, clean reads were aligned to the human genome (UCSC hg19) with Bowtie version 1.1.2, allowing only uniquely mapping reads with up to two mismatches. The aligned reads were normalized to total reads aligned (reads per million) using samtools. The track files were made with the bamCoverage command from deepTools 3.3.0. For normalization, the reads were aligned to the input group by Bowtie2 with a cutoff (q value < 0.1). Chip-seq peaks were called using MACS (model-based analysis of ChIP-seq) version 2.1.2 using default parameters and a q value cutoff of 1 × 10^–4^. The distribution of Chip-seq peaks was annotated with the R package ChIPseeker and visualized using IGV (v.2.8.2). Heatmaps, metaplots, and metagene plots were made for the indicated windows using deepTools 3.3.0. The different peaks between nutlin3 treated and nontreated groups were analyzed by R package DiffBind.

### 2.6 Cell viability assay for p53-mutant cell lines upon ATO treatment

36 cell lines harboring structural p53 mutants were seeded into 96-well plates with 30% confluence for 24 h, and there were three replicates for each sample. Diluted ATO was added to each well for 72 h incubation, and 10 μl of CCK-8 reagent (CK04, DOJINDO) was subsequently added into the plate. Plates were protected from light and incubated for 1 hour at 37 °C before the absorbance was detected at 450 nm on a Wallac EnVision plate reader (PerkinElmer). Readings of ATO-treated wells were normalized to untreated wells (set to 100% luminescence) and wells containing only media (set to 0% luminescence). IC_50_ values were calculated by GraphPad Prism 10.

### 2.7 p53 signature genes analysis

Mutations with a frequency > 0.5% and known to inactivate p53 transactivation activity, such as missense, frameshift, insertion, deletion, stop-gain, and splicing mutations, were used to define WT-UP genes. Similarly, high-frequency (> 0.5%) missense mutations with gain-of-function (GOF) properties were selected to define MIS-UP genes. WT-UP and MIS-UP genes were consistently identified across at least five TCGA cohorts. Patients lacking survival data were excluded from subsequent analysis. Differentially expressed genes were analyzed using the R package limma, which employs an empirical Bayesian method, with statistical significance defined as an adjusted *P*-value < 0.05 and log_2_FC > 0.58 (fold change > 1.5). For the survival analysis of p53 signature genes, patients were stratified into high- and low-expression groups based on the median expression level of the p53 signature genes within each cohort. HR were estimated using univariable Cox regression analysis, and statistical significance was assessed with the log-rank test. Three kinds of signature genes were performed Gene Ontology (GO) enrichment analysis using the R package clusterProfiler (v4.6.0) to interrogate the biological process (BP) ontology.

### 2.8 Transcriptome data collection

Transcriptome profiles of cell lines before and after treatment with p53 rescue compounds were collected from previous publications [24]. The expression profiles were generated using salmon (version 1.8.1). The human reference genome and annotations used were sourced from the GENCODE database (GRCh38, version 40, https://www.gencodegenes.org/). Salmon (version 1.8.1) was used to generate the count matrices and transcripts per kilobase of exon model per million mapped reads (TPM) matrix. Gene expression levels were evaluated using fragments per kilobase million (FPKM), calculated by normalizing gene lengths based on the TPM matrix. The FPKM matrix was log_2_-transformed as log_2_(FPKM + 1). A total of 18,296 genes present across all datasets were included for analysis. The transcriptome profiles were integrated and normalized using median normalization. Log_2_-transformed fold change (log_2_FC) was derived from the log_2_-transformed FPKM matrix. For identifying genes upregulated by ATO, the upregulated genes were calculated using log_2_FC between the sample treated by ATO and its untreated sample. Cutoff was set to log_2_FC > 0.58 (FC > 1.5) unless otherwise specified. Heatmaps were generated by the R package pheatmap (https://CRAN.R-project.org/package=pheatmap). For gene set enrichment analysis, the upregulated DEGs (GFOLD ≥1, log_2_FC ≥1.5) were input into Enrichr (https://amp.pharm.mssm.edu/Enrichr/), which returned the enriched terms.

### 2.9 Statistical analyses

Statistical analysis (unpaired two-tailed Student’s t-test, with a 95% confidence interval under the untested assumption of normality) was performed in GraphPad Prism 10. Data are presented as mean ± sd. Group size was indicated in the main text. Statistical details of experiments can be found in the Fig. Figs, legends, and results.

### 2.10 Data availability and code availability

The merged expression profiling of the transcriptome datasets was available at Supplement Table 6. The gene expression data of the TCGA Pan-Cancer cohorts included in this study can be accessed through the following link: https://xenabrowser.net/datapages/. The analysis code and data used for the study can be found at https://nrctm-bioinfo.github.io/p53_compounds.

## 3 Result

### 3.1 p53-related prognosis across 33 cancer types

**Fig. 1:**
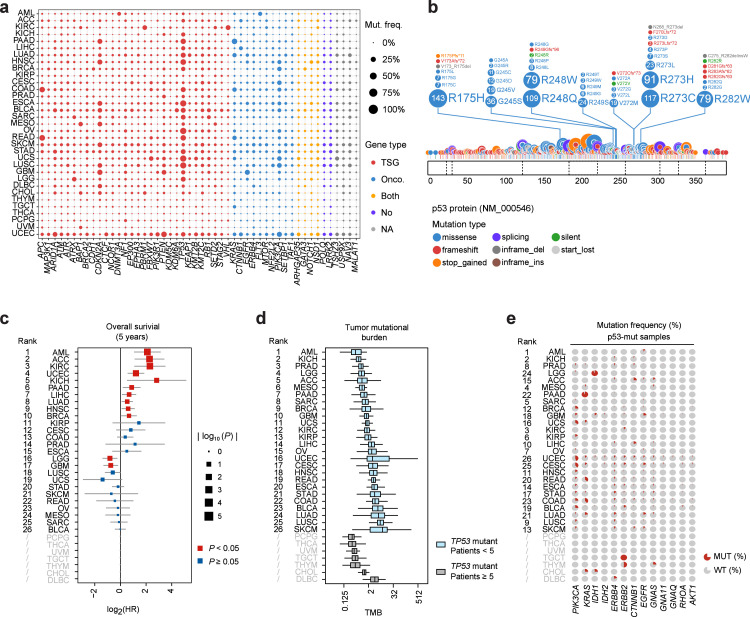
**p53-related prognosis across 33 cancer types**. (a) Dot plot of the mutation frequency of the indicated genes across the 33 cancer types, with data sourced from The Cancer Genome Atlas (TCGA) Pan-Cancer Atlas. The indicated genes are the top 50 out of 127 significantly mutated genes identified in 12 major cancer types [1]. Gene types are from OncoKB: TSG, tumor suppressor gene; Onco., oncogene. (b) Spectrum of *TP53* mutation with hotspot mutations labeled. (c) Distribution of hazard ratios (HR) and *P* value of p53 mutations for 5-year survival data across the 33 TCGA cancer type, the cancer types with *TP53* mutant patients < 5 were not analyzed and labeled as grey. (d) Distribution of tumor mutational burden across the 33 TCGA cancer types. The vertical lines within each bar represent the average TMB. (e) Pie charts showing the mutation frequencies of the indicated cancer driver genes, which are the top 13 mutated in pan-cancer [26], in the 33 TCGA cancers with p53 mutations. In (d-e), cancer types with TP53 mutant patients fewer than 5 were labeled in grey. AML, acute myeloid leukemia; ACC, adrenocortical carcinoma; KIRC, kidney renal clear cell carcinoma; KICH, kidney chromophobe; PAAD, pancreatic adenocarcinoma; LIHC, liver hepatocellular carcinoma; LUAD, lung adenocarcinoma; HNSC, head and neck squamous cell carcinoma; BRCA, breast invasive carcinoma; KIRP, kidney renal papillary cell carcinoma; CESC, cervical squamous cell carcinoma and endocervical adenocarcinoma; COAD, colon adenocarcinoma; PRAD, prostate adenocarcinoma; ESCA, esophageal carcinoma; BLCA, bladder urothelial carcinoma; SARC, sarcoma; MESO, mesothelioma; OV, ovarian serous cystadenocarcinoma; READ, rectum adenocarcinoma; SKCM, skin cutaneous melanoma; STAD, stomach adenocarcinoma; UCS, uterine carcinosarcoma; LUSC, lung squamous cell carcinoma; GBM, glioblastoma multiforme; LGG, brain lower grade glioma; DLBC, lymphoid neoplasm diffuse large B-cell lymphoma. CHOL, cholangiocarcinoma; THYM, thymoma; TGCT, testicular germ cell tumors; THCA, thyroid carcinoma; PCPG, pheochromocytoma and paraganglioma; UVM, uveal melanoma; UCEC, uterine corpus endometrial carcinoma.

Among the 127 significantly mutated genes across pan-cancer (Supplemental Table 1), the tumor suppressor gene (TSG) *TP53* is the most frequently mutated one [1]. It is mutated in about 42% of cancer cases, exhibiting a widespread mutation distribution across cancer types (Fig. 1a, showing the top 50 genes). *CDKN2A* and *PIK3CA* are the other two genes exhibiting widespread mutation across cancer types. This feature necessitates the selection of an appropriate cancer type in p53-rescue clinical trials.

To elucidate the impact of *TP53* mutations on the prognosis of various cancer types, we performed a systematic analysis using data from the Pan-Cancer Atlas of The Cancer Genome Atlas (TCGA), comprising 9875 cancer patients across 33 cancer types (Supplemental table 2). Many cancer types have > 0.5% *TP53* mutation frequency (Supplemental table 3). A variety of *TP53* mutation types are identified, including missense, frameshift, and splicing mutations (Fig. S1a, right panel). Notably, missense mutations predominate in 31 of 33 cancer types, accounting for more than half of all mutations. Missense mutations are the primary targets of the numerous mutant p53 rescue compounds. Further analysis of the *TP53* mutation spectrum revealed 6 mutational hotspots (R175, G245, R248, R249, R273, R282) as well as “warmspots” such as V272M (Fig. 1b).

We then evaluated the prognostic impact of *TP53* mutations across various cancer types; the cancer types with *TP53* mutant patients < 5 were labeled as grey in Fig. 1c–d. *TP53* mutations were most significantly associated with poorer prognosis in acute myeloid leukemia (AML, *P* = 2.2 × 10^–5^), adrenocortical carcinoma (ACC, *P* = 2.5 × 10^–5^), and kidney renal clear cell carcinoma (KIRC, *P* = 4.0 × 10^–5^) (Fig. 1c, survival curves shown in Fig. S1b). This suggests a crucial role of *TP53* mutation in driving disease progression and causing poor prognosis in these cancers. Further analysis of the tumor mutational burden (TMB) across cancer types revealed that AML had the lowest mutational burden, suggesting that *TP53* mutation is more likely an essential cancer driver event in AML (Fig. 1d). As cancer patients can harbor multiple gene mutations and these mutations may collectively drive disease progression, we next examined the concomitant mutations of the established cancer driver genes, which are the top 13 mutated genes in pan-cancer [26] with *TP53* mutation, such as mutations on *KRAS, PI3K*, and *EGFR*. In cancers with *TP53* mutation, such as low-grade glioma (LGG) or pancreatic adenocarcinoma (PAAD), a high frequency of *IDH1* or *KRAS* mutations was observed. However, in the subset of AML with *TP53* mutation, additional cancer driver mutations were rare or absent (Fig. 1e), implying that *TP53* mutation is likely the essential or sole driver event in the *TP53*-mutant AML patients.

Thus, we predict AML as the most sensitive cancer type to p53-rescue therapy, due to the most significant 5-year overall survival HR associated with *TP53* mutations, as well as the implication that *TP53* mutation is likely the essential or sole driver event in the subset of AML with *TP53* mutation.

### 3.2 Poor prognostic concordance in hematologic malignancies with p53 mutations in 126 cohorts

**Fig. 2:**
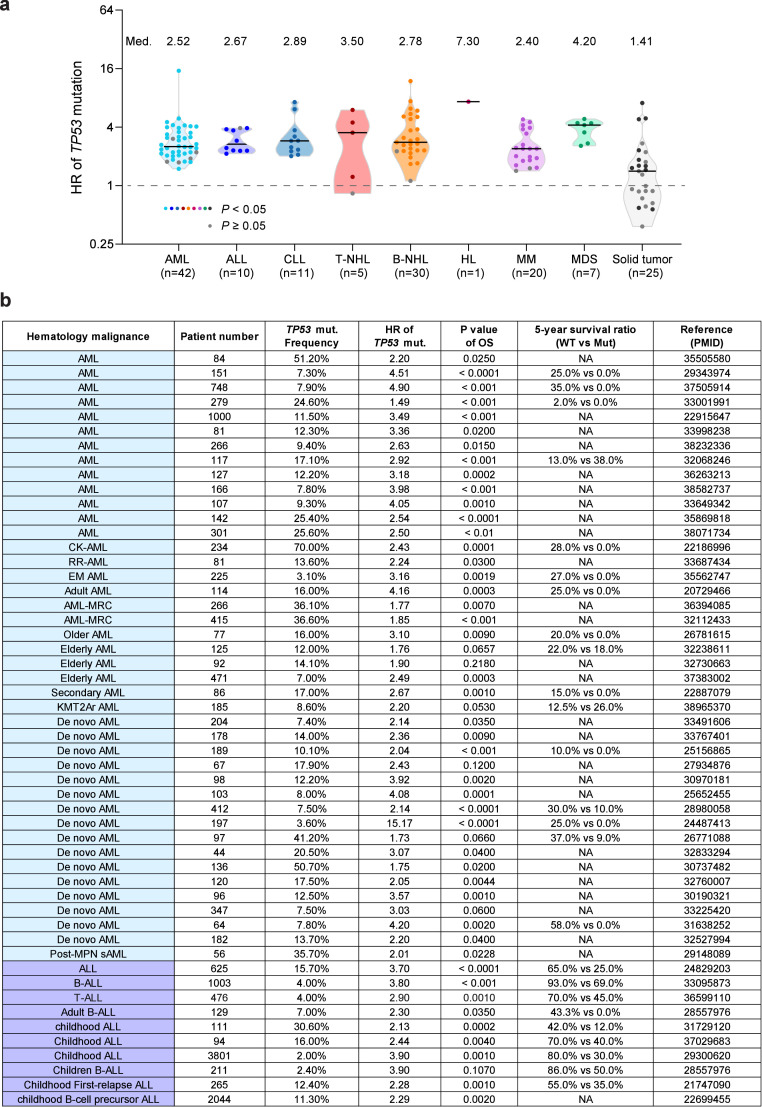

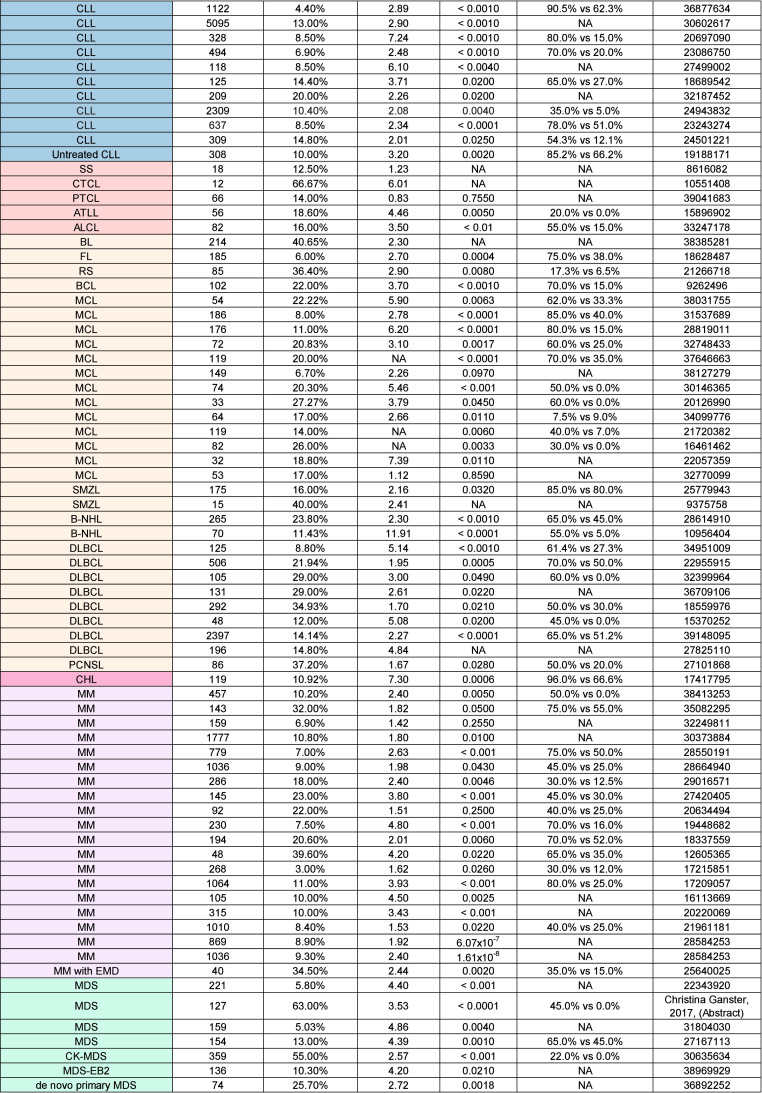
**Poor prognostic concordance in hematologic malignancies with p53 mutations in 126 cohorts**. (a) Violin plot summarizing the HR of *TP53* mutations in indicated hematological malignancies as reported in clinical studies, and in 25 solid tumor types from TCGA. Colorful circles represent significant HR (*P* < 0.05), while gray circles represent nonsignificant HR (*P* ≥ 0.05). (b) Table presenting the details from the clinical studies illustrated in (a). AML, acute myeloid leukemia; CK-AML, complex karyotype acute myeloid leukemia; RR-AML, relapsed/Refractory acute myeloid leukemia; EM AML, extramedullary acute myeloid leukemia; AML-MRC, acute myeloid leukemia with myelodysplasia-related changes; KMT2Ar AML, KMT2A-rearranged acute myeloid leukemia; Post-MPN sAML, post-myeloproliferative neoplasm secondary acute myeloid leukemia; ALL, acute lymphoblastic leukemia; B-ALL, B-cell acute lymphoblastic leukemia; T-ALL, T-cell acute lymphoblastic leukemia; CLL, chronic lymphocytic leukemia; SS, sezary syndrome; CTCL, Cutaneous T-cell lymphoma; PTCL, peripheral T-cell lymphoma; ATLL, adult T-cell leukemia/lymphoma; ALCL, anaplastic large cell lymphoma; BL, Burkitt lymphoma; FL, follicular lymphoma; RS, richter syndrome; BCL, B-cell lymphoma; MCL, mantle cell lymphoma; SMZL, Splenic marginal zone lymphoma; T-NHL, T-cell non-hodgkin lymphoma; B-NHL, B-cell non-hodgkin lymphoma; DLBCL, diffuse large B-cell lymphoma; PCNSL, primary central nervous system lymphoma; CHL, pediatric classical hodgkin lymphoma; MM, multiple myeloma; MM with EMD, multiple myeloma with extramedullary disease; MDS, myelodysplastic syndromes; CK-MDS, complex karyotype myelodysplastic syndromes; MDS-EB2, myelodysplastic syndromes with excess blasts-2.

AML is a subtype of hematologic malignancies, and we were asked whether p53 mutation consistently predicts poor prognosis in other hematologic malignancies. We thus reviewed 126 clinical studies with reported hazard ratios (HR) for *TP53* mutations in hematologic malignancies, including AML (42 studies), acute lymphocytic leukemia (ALL, 10 studies), chronic myelogenous leukemia (CML, 11 studies), chronic lymphocytic leukemia (CLL, 5 studies), T-cell or B-cell non-Hodgkin lymphoma (T-NHL/B-NHL, 5/30 studies), multiple myeloma (MM, 20 studies), and myelodysplastic syndrome (MDS, 7 studies) (Fig. 2a, details seen in Fig. 2b). In 125 of the 126 reviewed studies, *TP53* mutations are associated with poor outcomes (Fig. 2a, HR > 1). A T-NHL cohort is the only one exhibiting good prognosis for p53-mutant patients, yet it does not reach statistical significance (HR = 0.83, *P* = 0.75) [27]. Interestingly, a study involving 197 de novo AML patients reported an HR of 15.17 for p53 mutations (HR = 15.17, *P* < 0.001), possibly because only 7 patients had *TP53* mutations in this study [28]. Compared to the consistently high HR in hematological malignancies in the 125 studies, p53 mutations in solid tumors have a variable effect on survival, being associated with poor prognosis in 14 cancer types and with good prognosis in 11 cancer types (Fig. 2a, 25 TCGA cancer types with *TP53*-mutant patients > 5 were analyzed). Together, all types of hematological malignancies, not exclusive to AML, consistently exhibit poor prognosis for p53 mutations.

We further compared p53 mutations with other gene mutations commonly found in hematologic malignancies regarding their effects on prognosis. A study identified 23 commonly mutated genes in 200 de novo AML cases [29], including *DNMT3A, FLT3, NPM1, IDH1, IDH2*, and *TP53*. Notably, although these driver gene mutations have been shown to impact prognosis in large cohorts of AML and other hematologic malignancies [[30], [31], [32]], in this AML cohort, only *TP53* mutations demonstrated a statistically significant effect on overall survival (HR = 3.58, *P* = 1.01 × 10⁻⁷) [29]. A similar result was observed in another study of 156 AML patients [33]. In a study of 439 MDS patients, mutations in *EZH2, TP53, RUNX1, ASXL1,* and *ETV6* were significantly associated with poorer overall survival [34]. Once again, *TP53* mutations were linked to the worst prognosis, with a hazard ratio of 2.48. Similar results were observed in a cohort of 944 MDS patients [35]. In a study of 96 Natural Killer/T-cell lymphoma (NKTCL) patients, *TP53* and *DDX3X* were the two most frequently mutated genes, both showing deleterious effects [36]. Interestingly, *DDX3X* is an established transcriptional target of p53 [37], highlighting the importance of the p53-DDX3X axis in inhibiting this disease.

Together, the corresponding subtypes of hematological malignancies that exhibit a significantly poor prognosis for p53 mutations, not exclusive to AML, may be suitable cancer types for p53-rescue therapy.

### 3.3 Experimental validation of the sensitivity of hematological malignancy cells to the wild-type p53

Given the critical role of *TP53* mutation in driving hematological malignancies, we hypothesized that hematological malignancy cells would be sensitive to wild-type (WT) p53. To test this, we conducted data mining of cell proliferation upon treatment of WT-p53 function-boosting compounds (namely small-molecule antagonists of p53-inhibitory MDM2 and/or MDMX) [[38], [39], [40], [41], [42], [43], [44], [45]], followed by experimental validation.

**Fig. 3:**
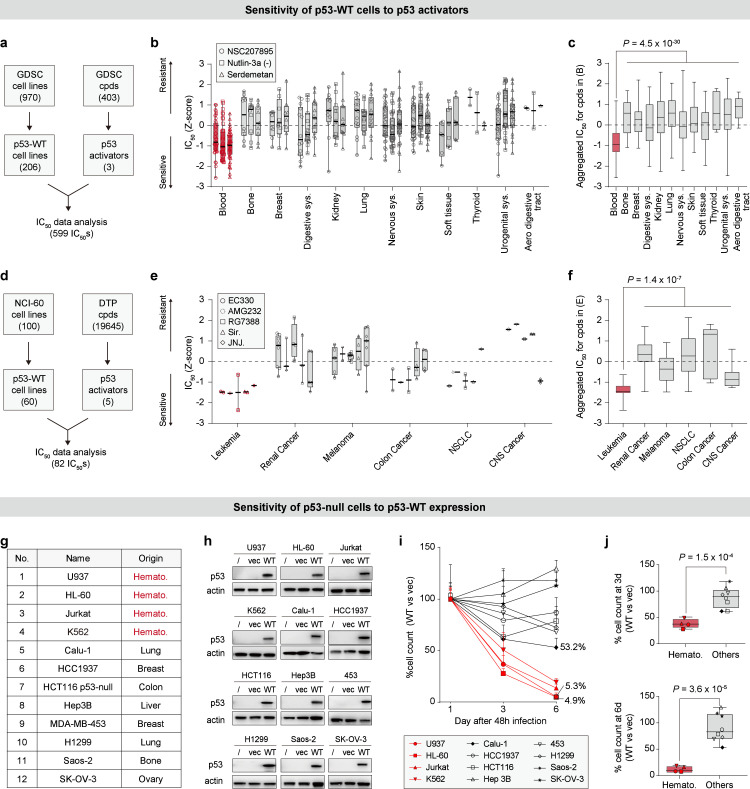
**Experimental validation of the sensitivity of hematological malignancy cells to the wild-type p53**. (a-f) Sensitivity analysis of wild-type p53 (p53-WT) cells to WT-p53 boosting compounds. (a,d) Flowcharts of the analysis process. The same pipeline of analysis was independently applied to the GDSC datasets and the NCI-60 screening database. (b,e) Box plots showing the IC_50_ values (Z-score) of the indicated WT-p53 boosting compounds for the cell lines derived from the indicated tissues or cancer types. The data were obtained from the GDSC datasets and NCI-60 screening data, respectively. Sys., system; sir, siremadlin; JNJ, JNJ-27291199. (c,f) Box plots showing the aggregated IC_50_ values (Z-score) for all WT-p53 boosting compounds in (b) or (e), respectively. (g-j) Sensitivity analysis of p53-null cells to p53-WT expression. (g) Characteristics of the cell lines used. Hemato., hematological malignancies. (h) Immunoblotting analysis of p53 expression levels at the starting point of the cell growth observation assay. The indicated cell lines were infected with the wild-type p53 (WT) or vector (vec) for 48 h, followed by immunoblotting determination. 453, MDA-MB-453. (i) Line graph showing the percentage of cell counts for the indicated cells infected with wild-type p53 compared to the vector-infected cells over the indicated days. Cell numbers were counted from three randomly picked scopes. (j) Box plot summarizing the percentage of cell counts at the indicated time point in (i), categorized by the presence of hematological malignancies. In (c), (f) and (j), error bars represent as means ± SD, *P* values were calculated using a two-tailed Student’s *t*-test.

We analyzed the sensitivity of various p53-WT cell lines to WT-p53 boosting compounds using two databases. The Genomics of Drug Sensitivity in Cancer (GDSC) database assessed the sensitivity of 970 cell lines to 403 compounds [46]. In this database, we identified 206 p53-WT cell lines derived from 12 different cancer types and 3 WT-p53 boosting compounds, obtaining a total of 599 IC_50_ values (Fig. 3a). Blood system-derived cell lines exhibited overall highest sensitivity to the three WT-p53 boosting compounds (Fig. 3b and S3a), significantly exceeding those observed in cell lines from other 11 tissues (Fig. 3c, *P* = 4.5 × 10^–30^). The NCI60 database assessed the sensitivity of 100 cell lines to 19,645 compounds [47]. In this database, we identified 60 p53-WT cell lines derived from 6 different cancer types and 5 WT-p53 boosting compounds, obtaining a total of 82 IC_50_ values (Fig. 3d). Again, leukemia cell lines exhibited highest sensitivity to these 5 WT-p53 boosting compounds (Fig. 3e and S3b), with sensitivity significantly higher than those observed in cell lines of other 5 cancer types (Fig. 3f, *P* = 1.4 × 10^–7^).

We next validated the sensitivity of p53-null cancer cells to the introduction of wild-type p53. A total of 12 p53-null cell lines derived from 7 different cancer types were collected, including 4 hematological malignancies and 8 solid tumor cell lines (Fig. 3g). We introduced wild-type p53 into these cell lines through viral infection, with vector-infected cells serving as an internal control (Fig. 3h). The results showed that all four hematological malignancy cell lines were more sensitive to wild-type p53 compared to the solid tumor cells (Fig. 3i and S3c, red vs black lines; representative cell morphology seen in Fig. S3d). On day 6 after infection of WT p53, the survival rates of U937 and HL-60 were only 5.3% and 4.9% of the corresponding lines infected with blank vector, respectively. In contrast, the survival rates of solid tumor cells remained above 50%. On both day 3 and day 6, hematological malignancy cell lines were significantly more sensitive to WT p53 than solid tumor cell lines (Fig. 3j, *P* = 1.5 × 10^–4^ and 3.6 × 10^–6^, respectively).

**Fig. 4:**
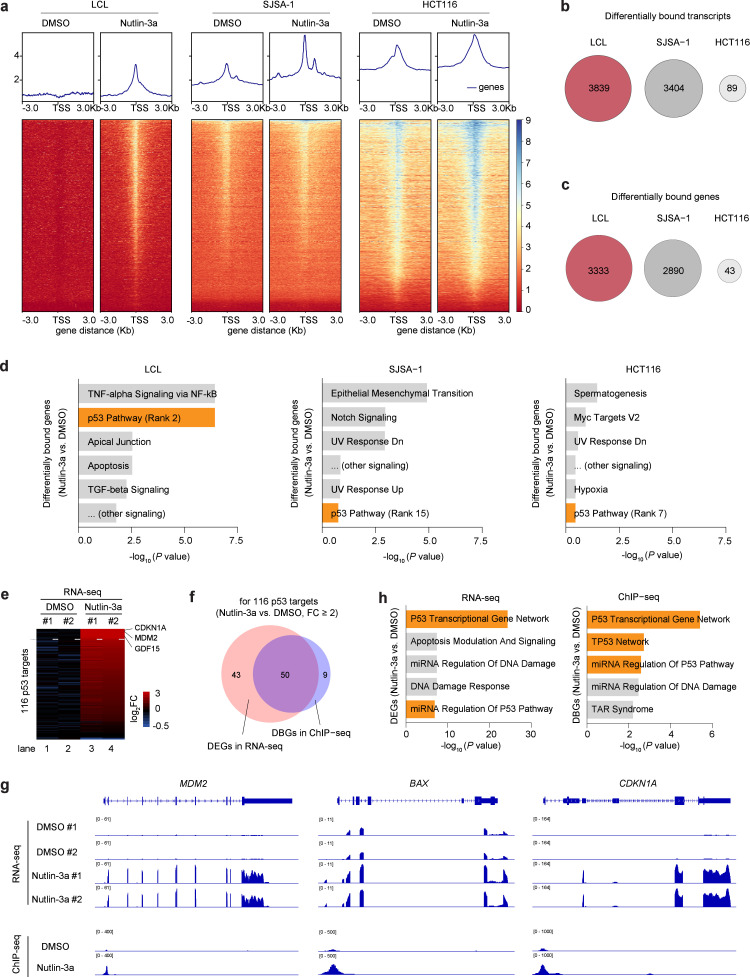
**p53 chromatin binding and target expression by ChIP-seq and RNA-seq**. (a) Aggregate metagene profiles (top) and heatmaps (bottom) of normalized ChIP-seq read density around transcription start sites (TSS ± 3 kb) in the indicated WT-p53 cell lines before (DMSO) and after (Nutlin-3a) treatment. (b-c) The number of differentially bound transcripts (b, FC ≥12) and differentially bound genes (c, FC ≥12) before (DMSO) and after (Nutlin-3a) p53 activation in each cell. (d) Enrichment analysis for the differentially binding genes against the MSigDB_Hallmark_2020 database, the “p53 pathway” was marked in orange. (e) Heatmap of 116 p53 targets in WT-p53 HCT116 cells in RNA-seq. (f) Venn diagram showing overlap between the differentially expressed genes (DEGs, from RNA-seq) and differentially bound genes (DBGs, from ChIP-seq) of the 116 p53 targets (FC ≥2, Nutlin-3a vs. DMSO). (g) UCSC Genome Browser views for the indicated p53 targets, with RNA-seq expression tracks (upper panels) and ChIP-seq occupancy tracks (lower panels). (h) Enrichment analysis for the DEGs in RNA-seq and DBGs in ChIP-seq against the Panther 2016 dataset, the p53-related pathway was marked in orange.

To investigate the mechanism underlying the differential sensitivity to wild-type p53 activation, we investigated the p53 binding DNAs through CHIP-seq in hematological LCL with solid-tumor SJSA-1 and HCT116, before (DMSO) and after (Nutlin-3a) p53 activation. Wild-type p53 in LCL exhibited a marked increase in DNA binding, whereas SJSA-1 and HCT116 showed only modest gains (Fig. 4a), despite higher basal occupancy (as indicated by stronger signals in the DMSO groups), supporting that these solid-tumor cells are less sensitive to growth suppression mediated by wild-type p53 activation. Consistently, LCL exhibited a much larger number of differentially bound transcripts and genes than SJSA-1 and HCT116 (Fig. 4b–c). Moreover, MSigDB Hallmark 2020 analysis revealed that DBGs in LCL were significantly enriched for the “p53 Pathway” (*P* = 3.67 × 10^–7^), compared with SJSA-1 and HCTA116 (*P* = 0.17 and 0.35, respectively) (Fig. 4d). Finally, the well-established p53 target genes—*MDM2, BAX, CDKN1A, TIGAR GADD45A, and PUMA* (*BBC3*)—showed greater ChIP signal enhancement in LCL than the other two solid-tumor cell lines upon Nutlin-3a treatment (Fig. S4a). Together, these results indicate that hematological LCLs exhibit stronger p53 DNA binding and stronger p53 target enrichment upon wild-type p53 activation compared with solid tumor cell lines.

To further integrate transcriptional changes with p53 binding, we further performed RNA-seq before (DMSO) and after (Nutlin-3a) treatment in HCT116 (Fig. 4e). Among the 116 p53 targets, RNA-seq identified 93 differentially expressed genes (DEGs; FC ≥2) and ChIP-seq identified 59 DBGs (FC ≥2) (Fig. 4f). The classic p53 targets showed strong concordance between mRNA upregulation and enriched promoter binding (Fig. 4g and S4b). Furthermore, both DEGs and DBGs were significantly enriched in p53 and p53-related pathways across multiple databases, including the Panther 2019 (Fig. 4h), NCI-Nature 2016 (Fig. S4c), and MSigDB Hallmark 2020 (Fig. S4d). In summary, these results demonstrate a clear concordance between p53 transcriptional activation and genomic binding, supporting the reliability of p53-mediated regulation of target genes.

Overall, hematological malignancy cells were more sensitive to active or reintroduced wild-type TP53 than solid tumor cells, likely because they exhibit lower basal p53 chromatin binding, allowing a broader transcriptional response upon p53 activation.

### 3.4 Experimental validation of the sensitivity of hematological malignancy cells to the ATO-rescued mutant p53

**Fig. 5:**
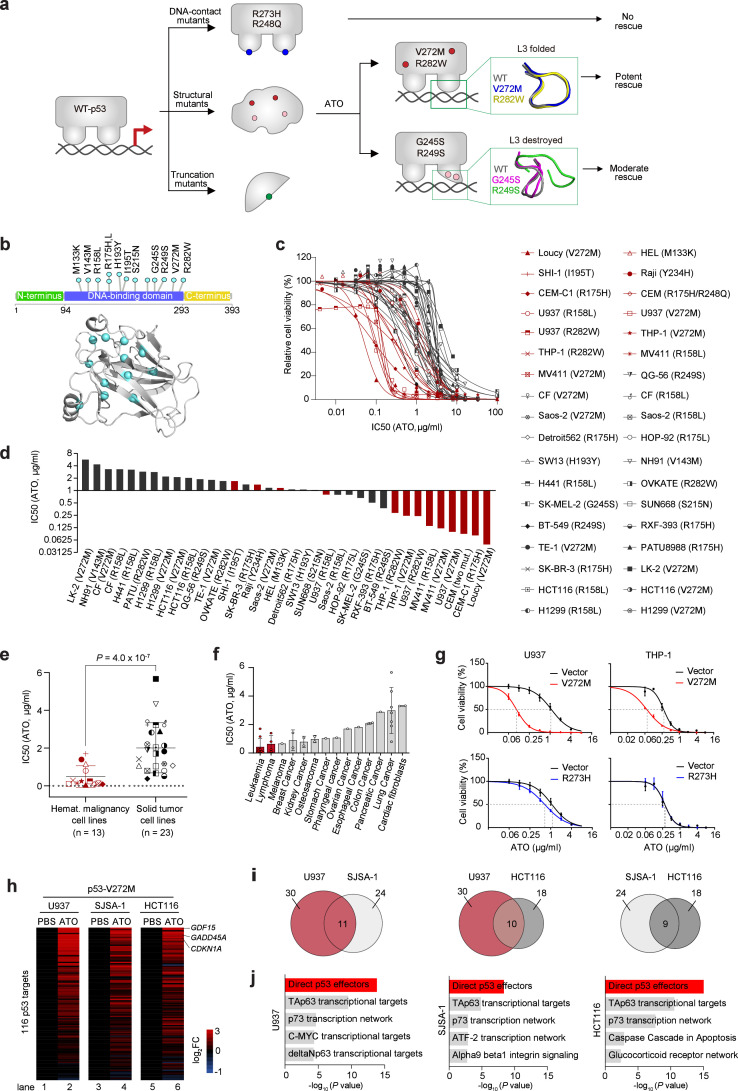
**Experimental validation of the sensitivity of hematological malignancy cells to the ATO-rescued mutant p53.** (a) Schematic model demonstrating why different mutants exhibit different sensitivity when rescued by ATO. (b) Upper panel: spectrum of *TP53* mutations present in the 36 cell lines. Lower panel: spatial locations of the *TP53* mutations in the 36 cell lines, as depicted in the upper legend, within the DNA binding domain (PDB; 1TUP). Alpha carbons of each mutated amino acid are shown as spheres. (c) Cell proliferation curves of the indicated cells treated with ATO at the indicated concentrations for 72 h. (d) Bar graph showing the calculated IC_50_ values of ATO for the indicated cell lines. (e) Box plot summarizing the IC_50_ values in (d), grouped based on whether the cell lines are derived from hematological malignancies. (f) Bar graph summarizing the IC_50_ values in (d), grouped by the derived cancer types of the respective cell lines. In (e) and (f), error bars represent means ± SD. *P* values were calculated using a two-tailed Student’s t-test. (g) U937 and THP-1 cells infected with the indicated p53 mutants were treated with ATO for 3 days, followed by cell viability determination. (h–j) p53-null cells expressing V272M were treated with PBS or ATO, followed by RNA-seq analysis. (h) Heatmap of the relative mRNA levels of the reported 116 p53 targets. (i) Venn diagram for the differentially expressed genes of the 116 p53 targets (FC ≥2) in the indicated cells. (j) Bar graphs showing the 5 most highly enriched pathways in the NCI-nature 2016 for the significantly differentially expressed genes (FC ≥2, GFOLD ≥1). The length of each bar represents the significance level of the enriched items.

p53 mutations can be briefly grouped into three classes (Fig. 5a): 1) DNA-contact mutations, which alter residues directly binding DNA (e.g., R273H, R248Q); 2) truncation mutations, which miss multiple residues; 3) structural mutations, promoting p53 unfolded [19]. ATO (and its analogs) have recently been reported as the only compound that can effectively rescue mutant p53, showing a preference toward structural mutants, across 10 widely used p53 assays [22]. Among structural mutants, one subset (e.g., V272M, R282W) undergoes complete global refolding upon ATO binding—fully restoring wild-type conformation to L3 DNA-binding loop and enabling potent rescue, whereas another subset (e.g., G245S, R249S) achieves global refolding but retains local disorder in L3 DNA-binding loop and confers only moderate rescue (Fig. 5a).

Next, we used ATO to assess the sensitivity of hematological malignancy cells to ATO-rescued mutant p53. A total of 13 blood cancer cell lines and 23 solid tumor cell lines were collected. Among these cell lines, the hematological malignancy cells U937, THP-1 and MV-411, as well as the solid tumor cell lines HCT116, H1299 and Saos-2, were initially p53-null and were stably infected with exogenously ATO-rescued mutant p53. Others harbored endogenous ATO-rescued mutant p53 [14,24]. Totally, these included 13 distinct structural mutations, including 5 hotspot and “warmspot” mutations (R175H, G245S, R249S, V272M, and R282W) (Fig. 5b, upper panel). The mutated amino acids are predominantly located in loop-sheet-helix (LSH) and β-sandwich domains, which are essential for p53’s structural stability (Fig. 5b, lower panel).

Among the 36 cancer cell lines, hematological malignancy cell lines overall exhibited greater cell death compared to solid tumor cell lines upon 72-hour ATO treatment (Fig. 5c, red curves shifted left compared to black curves). Based on the calculated half-maximal inhibitory concentrations (IC_50_), the 9 most sensitive cell lines were hematological malignancies (Fig. 5d; IC_50_ values for all cell lines shown in Fig. S5a). The ALL cell line Loucy, which harbors endogenous p53-V272M, had the lowest IC_50_ (Fig. 4c, <0.05 µg/ml). In contrast, the non-small cell lung cancer cell LK-2, with the same endogenous p53-V272M, had an extremely high IC_50_ (Fig. 5d, 5.7 µg/ml). Integration of the IC_50_ data revealed that hematological malignancy cell lines harboring structural p53 mutations were significantly more sensitive to ATO than solid tumor cell lines (Fig. 5e, median IC_50_ = 0.24 vs. 1.82 μg/ml, *P* = 4.0 × 10^–7^), with both leukemia and lymphoma cell lines being most sensitive (Fig. 5f, median IC_50_s ranked first and second, respectively).

To evaluate intracellular specificity of ATO, we selected the ATO-rescuable structural mutant V272M, and the unrescuable DNA-contact mutant R273H as a control. In the isogenic U937 and THP-1 model (without endogenous p53 expression) stably infected with vector, V272M and R273H, sharing an identical genetic background, ATO treatment selectively killed cells expressing V272M but not R273H or no p53 (Fig. 5g), confirming the specificity of ATO in targeting the cells harboring rescuable p53 mutant.

To further investigate the cell-type-dependent manner of ATO, we performed parallel RNA-seq in hematological malignancy cells (U937) and solid tumor cells (SJSA-1 and HCT116), all harboring exogenous p53-V272M. All three cells showed clear activation of the 116 well-established p53 targets following ATO treatment (lanes 2, 4, and 6 in Fig. 5h). Notably, the hematological cell line U937 showed relatively higher levels of induction. Quantitatively, U937 cells exhibited the strongest transcriptional response (30 genes with FC ≥2), followed by SJSA-1 (24 genes) and HCT116 (18 genes) (Fig. 5i). Moreover, DEGs in each cell line showed significant enrichment for the p53 pathway (Fig. 5j). Together, ATO treatment can effectively upregulate p53 target genes in cells expressing rescued mutant p53, and the extent is modulated in a cell-type–dependent manner.

In conclusion, hematological malignancy cells exhibited higher sensitivity to rescued mutant p53 than solid tumor cells.

### 3.5 Favorable change of transcriptome profile predicts efficacy of ATO in treating hematological malignancies

Our findings show that the p53-rescue compound ATO effectively induced cell death in p53-mutant hematological malignancies. Based on these results, we set out to preliminarily predict the potential efficacy of ATO in patients with hematological cancers according to ATO-induced transcriptome profile changes.

**Fig. 6:**
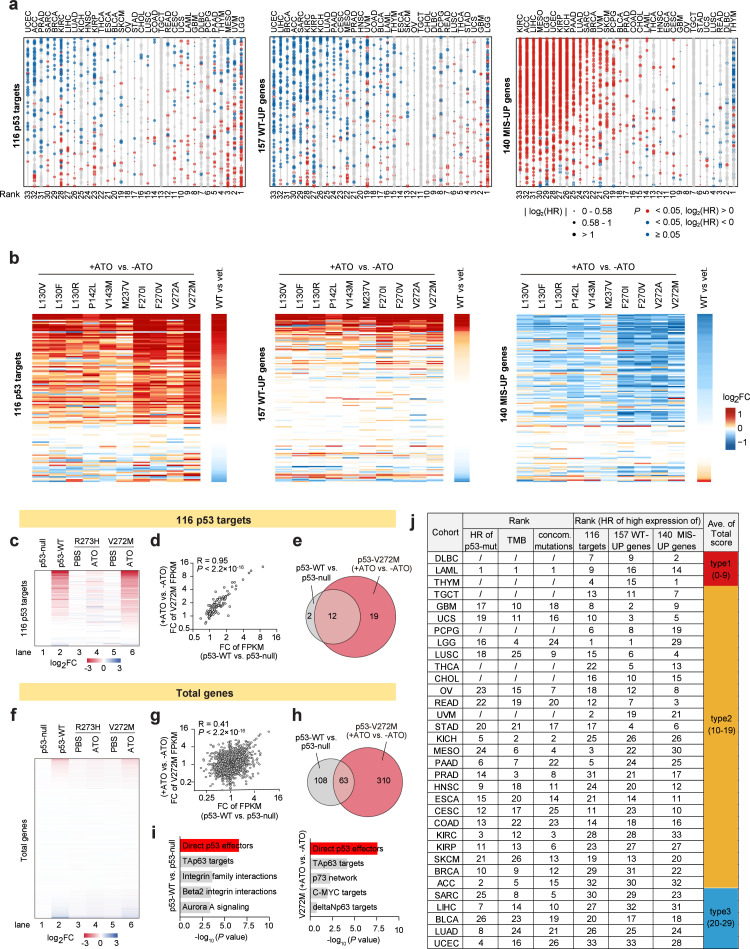
**Favorable change of transcriptome profile predicts efficacy of ATO in treating hematological malignancies**. (a) HR of high expression of p53 signature genes among TCGA cancer types. In each cohort, patients are separated based on the median value of gene expression. *P*-values are calculated using the log-rank test, and the HR values are calculated using the univariable Cox regression analysis. The favorable genes (*P*-value < 0.05, HR < 1) and unfavorable genes (*P*-value < 0.05, HR >1) in each cohort are marked as blue and red circles. (b) Heatmap of the log2-transformed fold change of the expression of p53 signature genes in hematological malignant U937 cell lines harboring indicated p53 mutations or wild-type p53 after ATO treatment. (c–i) U937 cells (p53-null) expressing WT-p53, R273H or V272M, were treated with PBS or ATO, followed by RNA-seq analysis. Heatmap of the relative mRNA levels of the reported 116 p53 targets (c) and total genes (f). Dot plots showed the fold change of relative mRNA levels for the reported 116 p53 targets (d) and total genes (g), comparing WT-p53 cells (relative to p53-null cells) with V272M cells (ATO treatment relative to PBS). Venn diagram showing overlap for the significantly differentially expressed (FC ≥2) in the 116 p53 targets (e) or whole genome (h). (i) Pathway enrichment analysis of the genome-wide differentially expressed genes in the NCI-nature database. (j) Ranked table summarizing six combined factors influencing cancer-type selection in p53-rescue clinical trials. Cancer types scoring 0–9 (red) are strongly recommended; 10–19 (orange) are conditionally recommended; 20–29 (blue) are not recommended.

First, we tried to define the lists of p53 signature genes whose expression levels are subject to regulation by p53. We searched for the genes with significant expression differences between wild-type p53 and p53-mutant patient tumors in the TCGA pan-cancer cohorts. Among 15,704 qualified genes in the TCGA pan-cancer cohorts (mean FPKM > 1), 157 genes showed significantly higher expression in tumors with wild-type p53 compared to those with p53 mutants (namely missense/frameshift/insertion/deletion/stop-gain/splicing mutations with > 0.5% mutation frequency), and these genes were designated as WT-UP genes (Fig. S6a, FC > 1.5, *P_adj_* < 0.05 in ≥5 TCGA cohorts; differentially expressed gene counts across cohorts are shown in Fig. S6b). Conversely, 140 genes exhibited significantly higher expression in tumors with p53 missense mutants (namely > 0.5% mutation frequency) compared to wild-type p53 tumors, and these genes were designated as MIS-UP genes (Fig. S6a, FC >1.5, *P_adj_* < 0.05 in ≥5 TCGA cohorts; differentially expressed gene counts across cohorts are shown in Fig. S6c). Together with the list of the 116 well-established p53 targets [48], we defined the three lists of p53 signature genes (Fig. 6a). In GO analysis, the 116 p53 targets and 157 WT-UP genes are enriched in tumor-suppressive pathways (p53 signaling, DNA damage response, apoptosis, G1/S transition checkpoint), whereas the 140 MIS-UP genes are involved in mitotic processes with no clear clinical benefit (Fig. S6d). These enriched terms are often involved in DNA lesion recognition and repair, enforcing cell-cycle fidelity, thereby regulating genomic stability [49,50], supporting the reported role of p53 in maintaining genomic instability [51].

HR analysis of high expression of p53 targets and WT-UP genes across the 33 cohorts revealed that these genes were overall associated with better prognosis (Fig. 6a, left and middle panel, more blue circles than red circles; detailed data in Supplement Table Tab 5). Conversely, MIS-UP genes exhibited the opposite impact, exhibiting overall poorer prognosis (Fig. 6a, right panel; more red circles than blue circles; detailed data in Supplement Table Tab 5). These findings suggest that the 116 established p53 targets, along with WT-UP and MIS-UP genes, may serve as potential prognostic markers for p53-related cancers.

Next, we performed transcriptomic analysis on isogenic hematological malignant U937 cell lines harboring the 10 p53 mutations that are reported to be most potently rescued by ATO [24] (Fig. 6b, and detailed expression profiling shown in Supplement Table Tab 6). The WT-p53 expressing U937 cell line was included as a reference. It turned out that ATO upregulated most of the 116 established p53 targets in all 10 cell lines, with the levels similar to those observed in WT cells compared to the vector control (Fig. 6b, left panel). As for the WT-UP and MIS-UP genes identified above, ATO overall upregulated WT-UP genes and downregulated MIS-UP genes, with changes comparable to those induced by WT expression (Fig. 6b, middle and right panels). Thus, ATO overall regulated the survival-beneficial p53 signature genes (116 p53 targets and WT-UP genes) and downregulated the survival-deleterious p53 signature genes (MIS-UP genes) in U937, both regulation patterns suggesting that ATO treatment may have therapeutic efficacy in treating hematological malignancies with p53 mutations. Subsequently, we generated volcano plots to show DEGs in each mutant line (Fig. S6e) and performed pathway enrichment analysis using the MSigDB Hallmark datasets (Fig. S6f). Remarkably, for 9 of the 10 mutants, the p53 pathway emerged as the top enriched term, including F270I, which showed the strongest enrichment (*P* = 5.65 × 10⁻¹⁸). These reflected consistent restoration of p53 transactivation activity by ATO across 10 structural mutants.

To further explore the specificity and efficacy of ATO to rescue structural p53 mutants, we performed RNA-seq in p53-null U937 cells engineered to express p53-WT, p53-V272M or p53-R273H. ATO treatment robustly activated 116 p53 targets in V272M cells to levels comparable with p53-WT cells, whereas R273H cells showed no obvious activation (Fig. 6c). The activation patterns between ATO-treated p53-V272M cells and p53-WT cells were almost identical (*R* = 0.95, *P* < 2.2 × 10–16, Fig. 6d), with 31 genes upregulated by ATO, 14 by WT-p53 overexpression, and 12 overlapped (Fig. 6e). Subsequently, genome-wide analysis revealed that the transcriptome of ATO-treated V272M cells closely mirrored that of p53-WT cells (Fig. 6f–g). Among the 373 genes induced by ATO and the 171 genes induced by WT-p53 overexpression, 63 genes overlapped between the two conditions (Fig. 6h). Notably, both gene sets showed significant enrichment for the p53 pathway (Fig. 6i). Taken together, these results demonstrate that ATO exerts its rescue effect primarily through p53-dependent transcriptional reactivation, closely recapitulating the gene expression program of wild-type p53.

## 4 Discussion and conclusion

The rationale for selecting cancer types is lacking in current p53-rescue trials. In a p53-rescue trial, it is not necessarily preferable to select cancer types with a high prevalence of p53 mutations, as a high mutation frequency may reflect that the mutation occurs at an early stage of cancer development rather than playing a cancer-driver role. For example, ovarian cancer exhibits a 93.38% p53 mutation frequency but has an HR of only 0.877 (Fig. 1c and Fig. S1b). Here, we predict hematological malignancy as the most likely responding cancer type to p53-rescue therapy based on large-scale TCGA data mining, literature review, and extensive experimental validation. It is consistent with the observations of *Trp53* genetically modified mice, wherein the hematological malignant lymphomas are the most frequently developed spontaneous tumor type [[52], [53], [54]]. In humans, cancer patients with Li-Fraumeni syndrome, a syndrome linked to germline mutation of p53, spontaneously develop ALL and lymphomas [55].

Surprisingly, when we assayed the prognosis of the three sets of p53 signature genes, we found that the 140 MIS-UP genes predominantly and profoundly predict poor prognosis (Fig. 5b right panel; the profile is predominantly red) whereas the p53 targets and WT-UP genes only partially and moderately predict beneficial prognosis (Fig. 5b left and middle panels; the profiles are overall blue but the blue is not predominant). This implies an underestimated role of mutant p53 GOF in determining patients' survival. Thus, abrogation of mutant p53 GOF may partially contribute to the tumor-suppressive function of ATO in p53-mutant cancers. Supporting this hypothesis, ATO exhibited satisfactory R175H-dependent tumor-suppressive effects in mouse studies [14,24] despite being far from fully restoring transactivation activity to p53-R175H [22]. The mechanism by which ATO abrogates the GOF of mutant p53 is unknown. It may be associated with the ability of ATO in abrogating structural instability of mutant p53 [14,56] since structural instability is thought to contribute to GOF phenotypes of p53 mutants [57]. We note that many groups have reported failure in observing the growth of function (GOF) of mutant p53 [[58], [59], [60]], and thus the existence of mutant p53 GOF and the current hypothesis of GOF abrogation by ATO need to be carefully investigated in future studies.

We therefore propose a data-driven framework for cancer type selection in p53-rescue trials, integrating six factors: HR of *TP53*-mutation, tumor mutational burden, co-mutation frequency, HR for high expression of the 116 p53 targets, the 157 WT-UP genes, and the 140 MIS-UP genes. Averaging each cancer type’s rank yields a composite score (Fig. 6j). This approach demonstrates DLBC, LAML and THYM as the top 3 candidates, among which DLBC and LAML both are hematologic malignancies, highlighting their potential as optimal candidates in the rapidly expanding p53-rescue clinical trials. The proposed cancer type selection guidelines for p53-rescue trials will undoubtedly help accelerate the achievement of statistically significant patient benefit in p53-rescue clinical trials in the future.

## CRediT authorship contribution statement

**Shujun Xiao:** Writing – review & editing, Writing – original draft, Project administration, Investigation, Data curation. **Jiaqi Wu:** Formal analysis. **Kai Tan:** Conceptualization, Formal analysis. **Sujiang Zhang:** Writing – original draft, Formal analysis. **Yuting Dai:** Investigation, Formal analysis. **Jiarui Wu:** Formal analysis. **Jiale Wu:** Funding acquisition, Formal analysis. **Xinle Han:** Formal analysis. **Derun Zheng:** Formal analysis, Data curation. **Xin Wang:** Formal analysis. **Huaxin Song:** Writing – review & editing, Writing – original draft, Funding acquisition. **Min Lu:** Writing – review & editing, Writing – original draft, Funding acquisition, Conceptualization.

## Declaration of competing interest

M. Lu and H. Song reports patents for ZL201880085409.4 (issued), ZL201980007369.6 (issued), and CN202110659578.8 (pending). M. Lu and S. Xiao reports a patent for ZL202110659536.4 (issued). No disclosures were reported by the other authors.

## Appendix Supplementary materials

Fig. S1. p53-related prognosis across 33 cancer types. (a) Overview of the sample counts, frequency of *TP53* mutations, and distribution of indicated mutation types of *TP53* in the 33 cancer types from the TCGA Pan-Cancer Atlas. (b) Five-year overall survival curves for patients with the indicated cancers. Patients were grouped based on the presence or absence of p53 mutations. Image, application 1
