# Supplementary material for: p53 mutation-associated prognosis across cancer types underlines hematological malignancy as an applicable cancer type to p53-rescue therapy
Source: Fundam Res. 2025 Jun 30;6(4):2711–24. doi: 10.1016/j.fmre.2025.06.011 (PMC13424698; doi:10.1016/j.fmre.2025.06.011)
Supplement: Supplementary file 1 — Fig. S1. p53-related prognosis across 33 cancer types. (a) Overview of the sample counts, frequency of TP53 mutations, and distribution of indicated mutation types of TP53 in the 33 cancer types from the TCGA Pan-Cancer Atlas. (b) Five-year overall survival curves for patients with the indicated cancers. Patients were grouped based on the presence or absence of p53 mutations. [file mmc1.pdf]

Figure S1

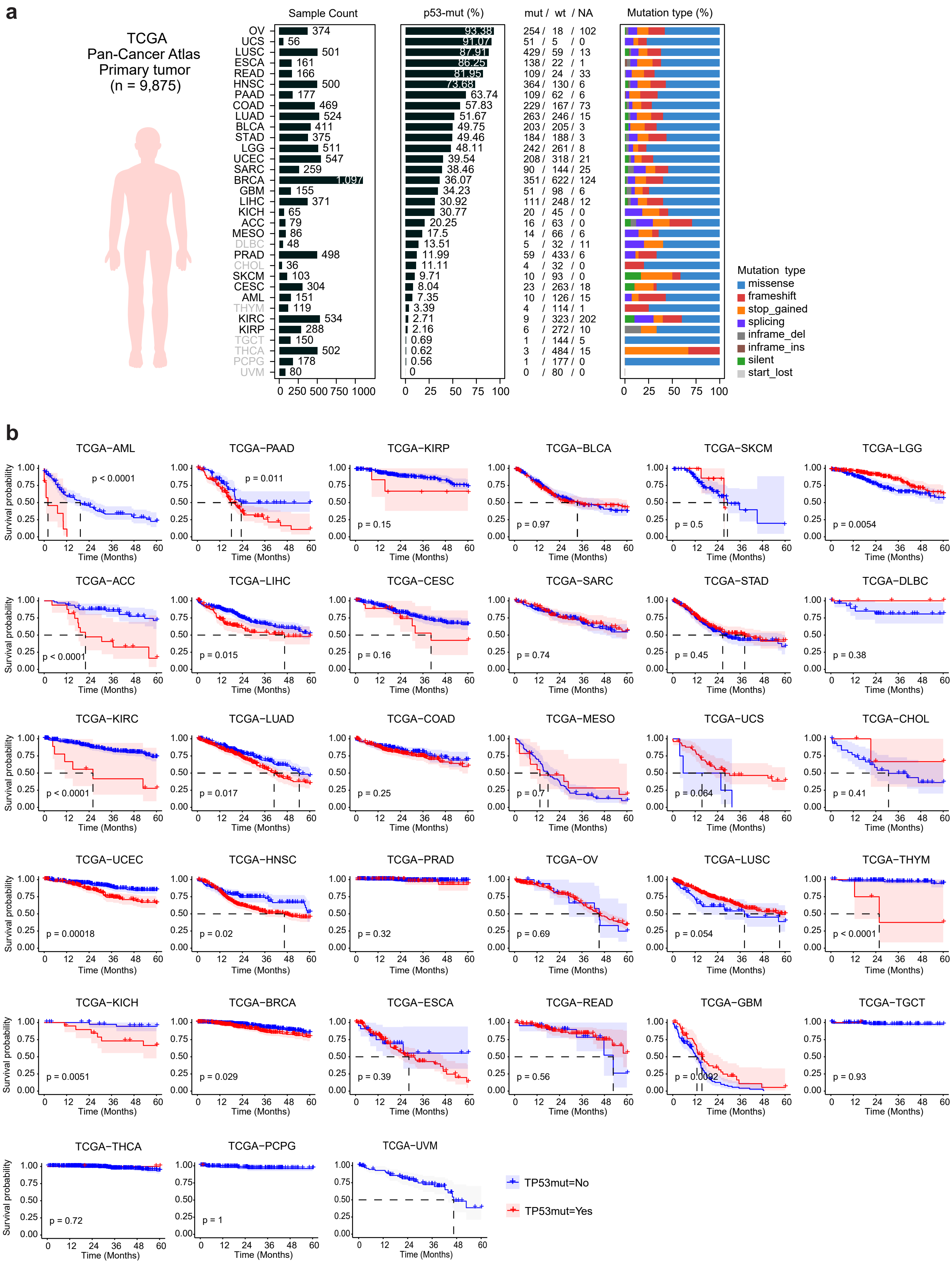
a
TCGA
Pan-Cancer Atlas
Primary tumor
(n = 9,875)
Sample Count
p53-mut (%)
mut / wt / NA
Mutation type (%)
Mutation type
missense
frameshift
stop_gained
splicing
inframe_del
inframe_ins
silent
start_lost
b
Survival probability
Time (Months)
TP53mut=No
TP53mut=Yes

Figure S3

a

NSC207895

Cell Line Name	IC50 (Z-score)	Cell Line Name	IC50 (Z-score)
JM1	-2.56	COLO-679	-0.04
NEC8	-2.46	MV-4-11	-0.03
G-401	-2.25	MS751	-0.01
HuTu-80	-2.01	NH-12	-0.01
BV-173	-2.00	NCI-H28	0.06
VA-ES-BJ	-1.97	NCI-H2052	0.11
CTB-1	-1.83	SW982	0.11
Mo-T	-1.80	LB1047-RCC	0.11
RF-48	-1.76	OVISE	0.12
MC-IXC	-1.71	CHP-126	0.16
NUGC-4	-1.54	ATN-1	0.16
KNS-81-FD	-1.51	G-402	0.19
IST-SL1	-1.44	BC-3	0.22
SK-HEP-1	-1.42	GAK	0.27
JAR	-1.40	OMC-1	0.31
A204	-1.34	KS-1	0.32
MEL-JUSO	-1.33	A101D	0.32
NALM-6	-1.32	JVM-3	0.35
QIMR-WIL	-1.29	COR-L105	0.35
SIG-M5	-1.29	ACHN	0.40
EoL-1-cell	-1.27	H4	0.41
MDST8	-1.25	GP5d	0.43
SR	-1.25	GI-ME-N	0.45
IGR-1	-1.23	KM-H2	0.49
BC-1	-1.21	HCC1500	0.49
RS4-11	-1.17	SCC-3	0.52
NB17	-1.16	CAL-54	0.52
YT	-1.11	HCT-116	0.53
KP-N-YN	-1.10	ME-180	0.54
MRK-nu-1	-1.06	CHP-212	0.58
697	-1.05	L-540	0.59
CADO-ES1	-1.01	DU-4475	0.63
CESS	-0.99	MDA-MB-175-VII	0.65
NKM-1	-0.99	CAL-51	0.66
OCI-AML2	-0.96	VMRC-MELG	0.68
UACC-257	-0.95	RMG-I	0.68
JSC-1	-0.94	MN-60	0.70
AGS	-0.92	MSTO-211H	0.71
HC-1	-0.92	LB2241-RCC	0.71
A3-KAW	-0.91	TOV-21G	0.72
Hey	-0.89	D-247MG	0.74
ECC10	-0.87	OS-RC-2	0.74
LC4-1	-0.83	L-428	0.76
BE-13	-0.81	LU-99A	0.77
CHP-134	-0.79	MES-SA	0.79
IMR-5	-0.77	MHH-NB-11	0.80
EW-3	-0.77	BB49-HNC	0.81
A375	-0.73	LS-513	0.83
NCI-H292	-0.71	SK-MEL-24	0.86
SW756	-0.70	HT-144	0.87
CP50-MEL-B	-0.67	A172	0.88
EB-3	-0.67	TE-12	0.89
MEL-HO	-0.67	OVTOKO	0.93
MHH-PREB-1	-0.67	ONS-76	0.95
Ca-Ski	-0.66	K5	0.98
RT4	-0.63	GRANTA-519	1.00
G-361	-0.63	JVM-2	1.01
ZR-75-30	-0.62	KU-19-19	1.02
769-P	-0.61	D-283MED	1.02
P30-OHK	-0.58	NCI-H460	1.02
A427	-0.57	NCI-H1563	1.03
GDM-1	-0.55	SK-MEL-1	1.04
NB12	-0.52	UACC-62	1.05
23132-87	-0.51	LoVo	1.06
NB1	-0.51	NB5	1.10
NB69	-0.50	LOXIMVI	1.10
AM-38	-0.48	NB10	1.17
MFH-ino	-0.48	LB996-RCC	1.21
WM-115	-0.44	U-2-OS	1.21
HT-1080	-0.42	HuO9	1.24
DoTc2-4510	-0.40	IST-MES1	1.27
H-EMC-SS	-0.40	SH-4	1.27
RPMI-8866	-0.40	HA7-RCC	1.27
HAL-01	-0.39	C3A	1.30
LB2518-MEL	-0.37	SW780	1.30
MZ7-mel	-0.34	A549	1.33
LB373-MEL-D	-0.33	SJSA-1	1.34
GOTO	-0.33	CP66-MEL	1.34
SF539	-0.31	RCC10RGB	1.35
SK-N-SH	-0.29	DBTRG-05MG	1.36
EW-7	-0.28	COLO-678	1.37
BPH-1	-0.24	COLO-792	1.37
MMAc-SF	-0.22	D-502MG	1.43
CTV-1	-0.21	SiHa	1.44
COLO-824	-0.19	LAN-6	1.47
OAW-42	-0.19	CAKI-1	1.49
A2780	-0.18	NCI-H1666	1.51
ES4	-0.16	NY	1.51
SK-MEL-5	-0.16	SKG-IIIa	1.57
UACC-812	-0.12	SW1573	1.60
SIMA	-0.09	CAL-72	1.67
DK-MG	-0.07	D-392MG	1.75
RPMI-6666	-0.07	TT	1.77
MZ2-MEL	-0.07	A498	1.88
NMC-G1	-0.07	C32	1.89
COLO-829	-0.05		

Serdemetan

Cell Line Name	IC50 (Z-score)	Cell Line Name	IC50 (Z-score)
NKM-1	-2.52	LOXIMVI	0.00
EoL-1-cell	-2.37	A498	0.02
MC-IXC	-2.21	SK-N-SH	0.02
BV-173	-2.07	LB2518-MEL	0.02
HC-1	-2.03	NCI-H2052	0.03
MV-4-11	-2.01	WM-115	0.03
JVM-2	-1.88	MEL-JUSO	0.04
JVM-3	-1.80	HCT-116	0.06
SR	-1.61	CHP-126	0.06
NCI-H460	-1.61	A172	0.07
BONNA-12	-1.57	SCC-3	0.08
OCI-AML2	-1.52	LB2241-RCC	0.08
MHH-CALL-4	-1.49	KP-N-RT-BM-1	0.08
HuTu-80	-1.44	A101D	0.08
CRO-AP2	-1.42	H-EMC-SS	0.09
NB69	-1.39	MFH-ino	0.11
JAR	-1.36	Ca-Ski	0.11
DOHH-2	-1.34	ES4	0.13
NB1	-1.30	TE-441-T	0.15
A3-KAW	-1.29	K5	0.16
IM-9	-1.23	NB17	0.17
MZ7-mel	-1.22	IST-MES1	0.18
BC-3	-1.19	A204	0.20
MM1S	-1.19	SIMA	0.20
JM1	-1.15	GOTO	0.23
RPMI-8866	-1.15	SF539	0.23
BC-1	-1.13	NB5	0.27
G-401	-1.13	ZR-75-30	0.28
DU-4475	-1.12	MN-60	0.32
EW-7	-1.07	LB1047-RCC	0.32
RS4-11	-1.02	OVTOKO	0.32
RPMI-6666	-1.01	GP5d	0.37
NB12	-0.98	NCI-H292	0.37
QIMR-WIL	-0.97	BPH-1	0.40
CTV-1	-0.97	VMRC-MELG	0.40
G-361	-0.92	COLO-792	0.42
CTB-1	-0.91	OVISE	0.42
H4	-0.89	SK-MEL-1	0.43
D-283MED	-0.89	AGS	0.48
MHH-PREB-1	-0.88	A375	0.50
GRANTA-519	-0.86	NCI-H1563	0.55
CADO-ES1	-0.85	A549	0.55
LAN-6	-0.85	C32	0.55
A2780	-0.82	KGN	0.56
BE-13	-0.78	Mo-T	0.59
JSC-1	-0.78	D-502MG	0.59
ATN-1	-0.78	UACC-812	0.62
HAL-01	-0.76	CAL-51	0.64
697	-0.76	U-2-OS	0.65
SW982	-0.73	MS751	0.67
IST-SL1	-0.71	LB373-MEL-D	0.69
MEL-HO	-0.69	DK-MG	0.71
G-402	-0.68	23132-87	0.74
L-540	-0.67	TOV-21G	0.74
RF-48	-0.60	LU-99A	0.75
MZ2-MEL	-0.59	GI-ME-N	0.76
SK-MEL-5	-0.56	SK-MEL-31	0.77
KU-19-19	-0.56	Hey	0.77
L-428	-0.52	SNG-M	0.86
SH-4	-0.52	CAL-72	0.87
MDST8	-0.51	CAKI-1	0.93
SK-MEL-24	-0.50	D-392MG	0.94
A427	-0.50	LB996-RCC	0.96
LC4-1	-0.49	SiHa	0.96
NALM-6	-0.48	TE-12	0.97
GDM-1	-0.46	RT4	0.99
NH-12	-0.45	HCC1500	1.03
P30-OHK	-0.42	HT-144	1.03
MES-SA	-0.42	LoVo	1.05
ACHN	-0.40	SW780	1.05
SIG-M5	-0.40	SJSA-1	1.06
EW-3	-0.39	KS-1	1.07
COLO-824	-0.38	SK-HEP-1	1.11
CHP-212	-0.38	MSTO-211H	1.17
CAL-54	-0.36	COLO-678	1.23
SK-CO-1	-0.35	OS-RC-2	1.25
769-P	-0.33	KNS-81-FD	1.25
CESS	-0.32	NY	1.25
CP50-MEL-B	-0.31	D-247MG	1.27
HA7-RCC	-0.31	ME-180	1.27
COLO-679	-0.31	DoTc2-4510	1.28
MHH-NB-11	-0.25	UACC-257	1.30
EB-3	-0.25	NCI-H1666	1.31
GAK	-0.24	ONS-76	1.35
D-245MG	-0.23	HT-1080	1.40
IGR-1	-0.20	CP66-MEL	1.42
KM-H2	-0.20	COLO-829	1.46
TT	-0.18	MMAc-SF	1.54
NCI-H929	-0.16	NCI-H28	1.54
HuO9	-0.14	C3A	1.64
UACC-62	-0.10	VA-ES-BJ	1.64
NB10	-0.09	SKG-IIIa	1.73
LS-513	-0.06	SW1573	1.76
KP-N-YN	-0.06	NUGC-4	1.85
MRK-nu-1	-0.06	NMC-G1	1.88
IMR-5	-0.06	COR-L105	2.04
ECC10	-0.06	RMG-I	2.07
YT	-0.05	MDA-MB-175-VII	2.19
OMC-1	-0.04	SW756	2.27
AM-38	-0.04	RCC10RGB	2.57
NEC8	-0.03	DBTRG-05MG	2.66

Nutlin-3a (-)

Cell Line Name	IC50 (Z-score)	Cell Line Name	IC50 (Z-score)
JVM-3	-1.80	HCC1500	-0.13
SR	-1.78	D-392MG	-0.11
DOHH-2	-1.74	RT4	-0.10
MV-4-11	-1.72	KU-19-19	-0.10
CRO-AP2	-1.71	KP-N-YN	-0.08
BC-1	-1.69	MZ7-mel	-0.07
NKM-1	-1.63	D-245MG	-0.05
LC4-1	-1.53	D-502MG	-0.05
HC-1	-1.52	NCI-H2052	-0.03
IM-9	-1.52	CAL-51	0.00
RPMI-6666	-1.43	TOV-21G	0.00
OCI-AML2	-1.41	Hey	0.02
BV-173	-1.41	WM-115	0.03
HuTu-80	-1.36	SNG-M	0.04
697	-1.36	LB2518-MEL	0.05
CHP-126	-1.34	Mo-T	0.09
GDM-1	-1.31	GI-ME-N	0.11
EoL-1-cell	-1.31	A204	0.13
BONNA-12	-1.30	CAKI-1	0.15
A3-KAW	-1.29	C3A	0.21
RS4-11	-1.29	HA7-RCC	0.24
H4	-1.28	IST-MES1	0.24
JVM-2	-1.28	LB373-MEL-D	0.27
CESS	-1.22	UACC-812	0.27
NEC8	-1.19	COR-L105	0.30
DU-4475	-1.12	BE-13	0.34
NALM-6	-1.11	KNS-81-FD	0.34
CHP-134	-1.11	MEL-HO	0.37
SJSA-1	-1.09	SIG-M5	0.38
SW982	-1.08	OVTOKO	0.39
JSC-1	-1.07	UACC-62	0.44
NCI-H460	-1.06	SH-4	0.49
NB17	-1.06	ES4	0.50
MHH-CALL-4	-1.06	SW780	0.51
MHH-NB-11	-1.03	SK-MEL-24	0.54
TE-441-T	-1.03	NB5	0.56
HAL-01	-1.02	EB-3	0.57
DK-MG	-1.01	OVISE	0.60
NCI-H929	-1.01	SK-HEP-1	0.60
IMR-5	-1.01	MEL-JUSO	0.61
LoVo	-0.96	LU-99A	0.61
QIMR-WIL	-0.95	SCC-3	0.61
CAL-54	-0.95	AM-38	0.63
RPMI-8866	-0.93	IST-SL1	0.63
L-540	-0.89	MC-IXC	0.64
MZ2-MEL	-0.87	NB1	0.69
KP-N-RT-BM-1	-0.84	NCI-H28	0.70
CADO-ES1	-0.83	NCI-H1666	0.70
A2780	-0.82	RCC10RGB	0.71
GP5d	-0.78	NCI-H1563	0.72
KGN	-0.76	A498	0.72
NB69	-0.76	EW-3	0.73
SK-CO-1	-0.75	MFH-ino	0.76
LS-513	-0.72	GAK	0.78
NH-12	-0.72	COLO-792	0.79
BC-3	-0.70	MHH-PREB-1	0.81
NB10	-0.70	MDA-MB-175-VII	0.83
CHP-212	-0.69	VA-ES-BJ	0.84
YT	-0.64	CAL-72	0.84
CTV-1	-0.64	COLO-679	0.85
MM1S	-0.63	U-2-OS	0.86
SK-N-SH	-0.63	MMAc-SF	0.91
JAR	-0.62	H-EMC-SS	0.95
RF-48	-0.62	G-361	0.99
G-401	-0.62	LB2241-RCC	1.00
LAN-6	-0.60	HuO9	1.01
D-283MED	-0.60	ONS-76	1.10
G-402	-0.60	ATN-1	1.10
LB996-RCC	-0.58	C32	1.13
P30-OHK	-0.56	UACC-257	1.14
ACHN	-0.56	L-428	1.17
SIMA	-0.53	SF539	1.18
AGS	-0.52	HT-1080	1.21
LOXIMVI	-0.51	OAW-42	1.24
MRK-nu-1	-0.51	ECC10	1.25
CP50-MEL-B	-0.50	NY	1.26
CTB-1	-0.50	IGR-1	1.28
GOTO	-0.49	MSTO-211H	1.29
A375	-0.48	ME-180	1.39
HCT-116	-0.44	SW1573	1.42
A172	-0.44	MS751	1.42
A549	-0.43	COLO-824	1.43
769-P	-0.42	SK-MEL-1	1.44
VMRC-MELG	-0.42	SK-MEL-5	1.46
JM1	-0.39	NUGC-4	1.47
D-247MG	-0.38	COLO-678	1.48
NCI-H292	-0.37	DBTRG-05MG	1.50
NB12	-0.37	TT	1.60
MES-SA	-0.36	NMC-G1	1.60
K5	-0.34	TE-12	1.60
GRANTA-519	-0.30	OMC-1	1.62
EW-7	-0.29	LB1047-RCC	1.64
MN-60	-0.29	Ca-Ski	1.68
MHH-CALL-2	-0.28	SiHa	1.75
KS-1	-0.27	ZR-75-30	1.77
MDST8	-0.26	COLO-829	1.80
KM-H2	-0.24	OS-RC-2	2.02
HT-144	-0.24	DoTc2-4510	2.07
A427	-0.23	CP66-MEL	2.08
23132-87	-0.22	BPH-1	2.14
A101D	-0.21	RMG-I	2.18
SK-MEL-31	-0.15	SKG-IIIa	2.21
BB49-HNC	-0.15	SW756	2.27

b

AMG-232

Cell Line Name	IC50 (Z-score)
SR	-1.54
NCI-H460	-1.01
HCT-116	-0.51
CAKI-1	-0.42
ACHN	-0.23
LOXIMVI	0.05
SK-MEL-5	0.69
A498	1.15
SF539	1.82

JNJ-27291199

LOXIMVI	-1.46
CAKI-1	-1.46
SR	-1.16
SF539	-1.03
UO31	-1.03
ACHN	-1.01
SF539	-0.85
A549	-0.46
A549	-0.33
UACC-62	-0.20
A498	0.50
CAKI-1	0.52
NCI-H460	0.55
NCI-H460	0.58
HCT-116	0.60
SK-MEL-5	0.67
UACC-257	1.01
SK-MEL-5	1.22
UACC-257	1.65
UACC-257	1.71

RG-7388

SR	-2.36
NCI-H460	-1.47
HCT-116	-1.27
SR	-0.64
HCT-116	-0.62
A549	-0.32
LOXIMVI	-0.01
UO31	0.05
LOXIMVI	0.10
UACC-62	0.22
SK-MEL-5	0.31
UACC-62	0.32
UACC-257	0.35
UACC-257	0.45
A498	0.81
CAKI-1	0.85
SF539	1.10
A498	2.14

Siremadlin

SR	-1.49
SR	-1.46
CAKI-1	-1.02
HCT-116	-0.98
NCI-H460	-0.82
LOXIMVI	-0.62
NCI-H460	-0.38
A549	-0.19
ACHN	-0.18
LOXIMVI	0.17
UACC-62	0.82
A549	0.92
SK-MEL-5	1.24
A498	1.31
SF539	1.33
SF539	1.34

EC-330

SR	-1.50
SR	-1.46
NCI-H460	-1.40
HCT-116	-1.18
LOXIMVI	-0.79
ACHN	-0.72
LOXIMVI	-0.51
A549	-0.36
CAKI-1	-0.33
ACHN	-0.25
SK-MEL-5	0.18
SK-MEL-5	0.57
CAKI-1	0.67
UACC-257	0.82
UO31	0.90
UO31	1.12
A498	1.30
A498	1.37
SF539	1.56

c

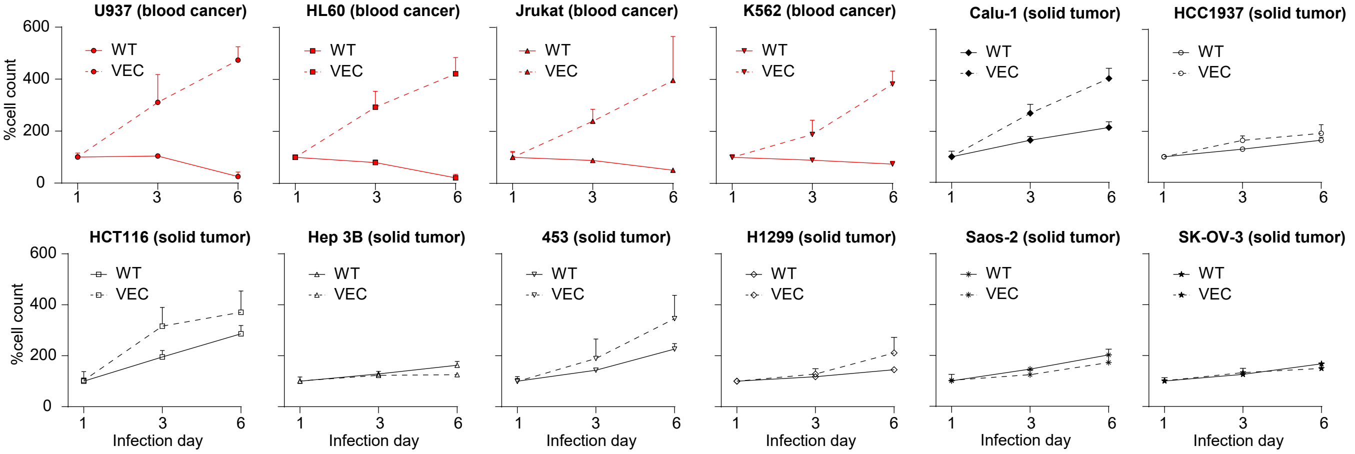
U937 (blood cancer)
HL60 (blood cancer)
Jrukat (blood cancer)
K562 (blood cancer)
Calu-1 (solid tumor)
HCC1937 (solid tumor)
HCT116 (solid tumor)
Hep 3B (solid tumor)
453 (solid tumor)
H1299 (solid tumor)
Saos-2 (solid tumor)
SK-OV-3 (solid tumor)
WT
VEC
%cell count
0
200
400
600
1
3
6
Infection day

d

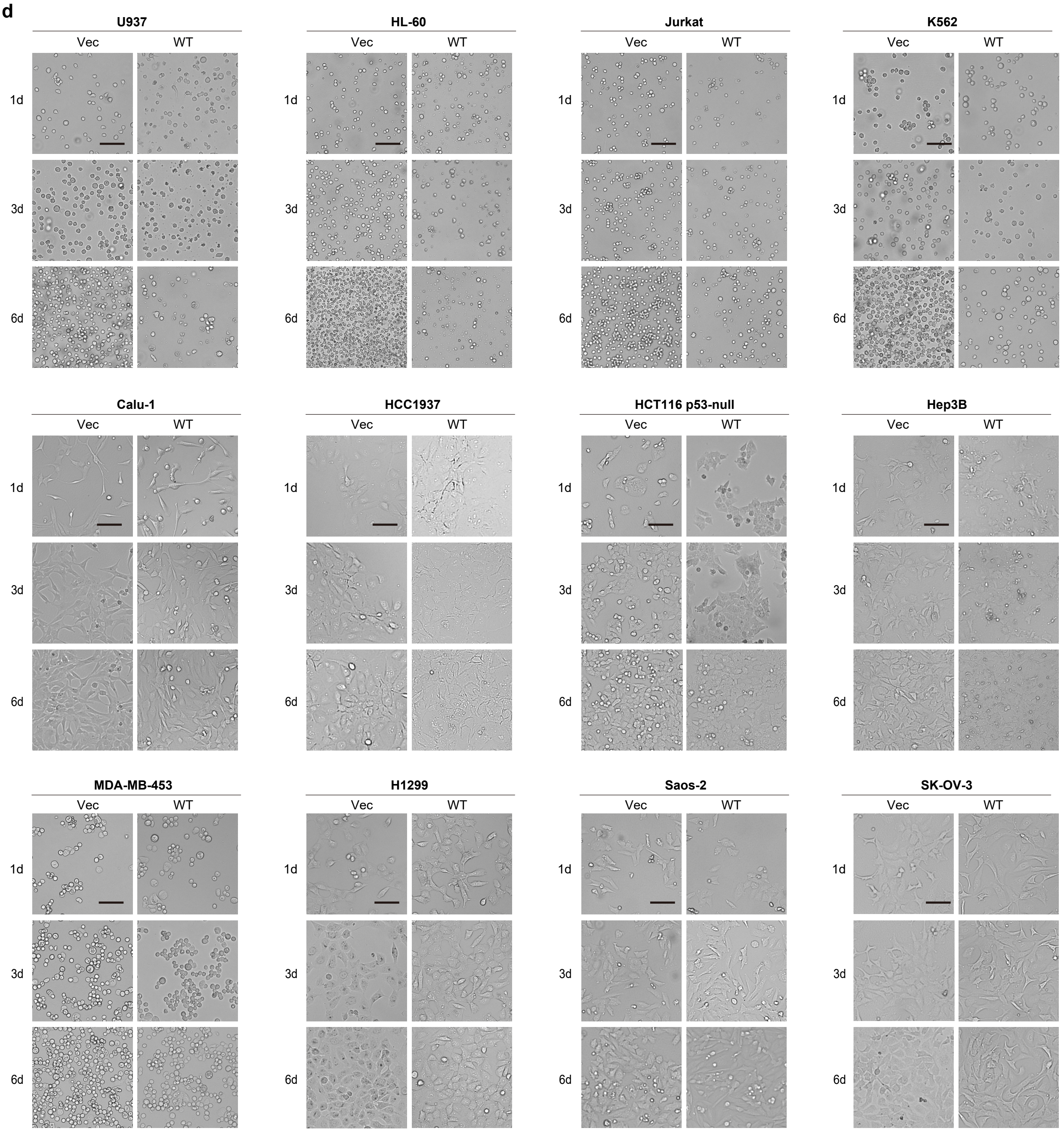
U937
HL-60
Jurkat
K562
Calu-1
HCC1937
HCT116 p53-null
Hep3B
MDA-MB-453
H1299
Saos-2
SK-OV-3
Vec
WT
1d
3d
6d

Figure S4

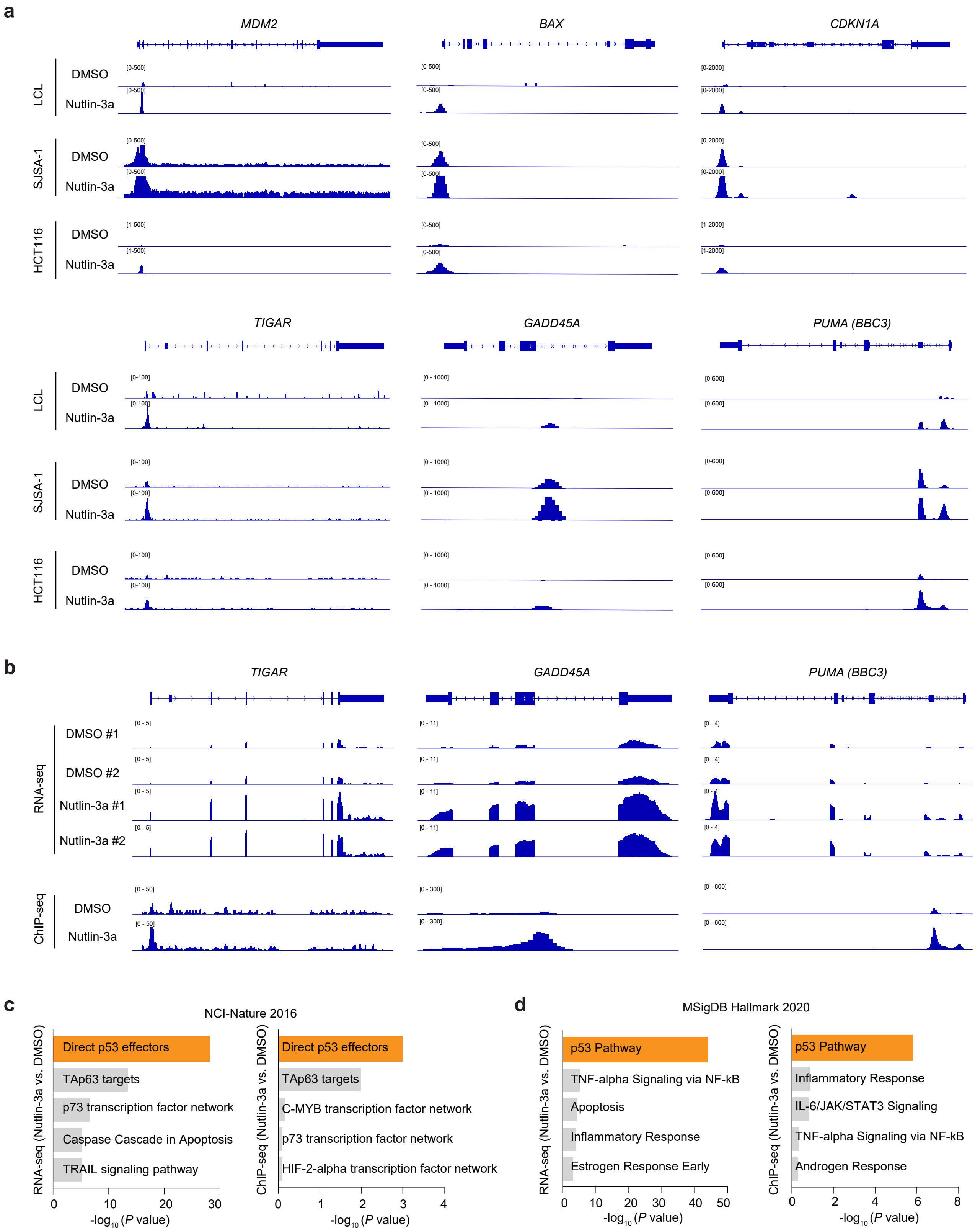
a
MDM2
BAX
CDKN1A
LCL
SJSA-1
HCT116
DMSO
Nutlin-3a
TIGAR
GADD45A
PUMA (BBC3)
b
RNA-seq
DMSO #1
DMSO #2
Nutlin-3a #1
Nutlin-3a #2
ChIP-seq
c
NCI-Nature 2016
RNA-seq (Nutlin-3a vs. DMSO)
Direct p53 effectors
TAp63 targets
p73 transcription factor network
Caspase Cascade in Apoptosis
TRAIL signaling pathway
ChIP-seq (Nutlin-3a vs. DMSO)
C-MYB transcription factor network
HIF-2-alpha transcription factor network
-log10 (P value)
d
MSigDB Hallmark 2020
p53 Pathway
TNF-alpha Signaling via NF-kB
Apoptosis
Inflammatory Response
Estrogen Response Early
IL-6/JAK/STAT3 Signaling
Androgen Response

Figure S5

a

Cell line	p53 status	Cell Type	Cancer Type	ATO IC50 μg/ml
Loucy	V272M	Hematological malignancy cell lines	leukaemia	0.049
CEM-C1	R175H	Hematological malignancy cell lines	leukaemia	0.081
CCRF-CEM	R175H/R248Q	Hematological malignancy cell lines	leukaemia	0.089
U937	V272M	Hematological malignancy cell lines	lymphoma	0.099
MV411	V272M	Hematological malignancy cell lines	leukaemia	0.121
MV411	R158L	Hematological malignancy cell lines	leukaemia	0.138
U937	R282W	Hematological malignancy cell lines	lymphoma	0.241
THP-1	V272M	Hematological malignancy cell lines	leukaemia	0.244
THP-1	R282W	Hematological malignancy cell lines	leukaemia	0.287
BT-549	R249S	Solid tumor cell lines	Breast Cancer	0.373
RXF-393	R175H	Solid tumor cell lines	Kidney cancer	0.508
SK-MEL-2	G245S	Solid tumor cell lines	Melanoma	0.661
HOP-92	R175L	Solid tumor cell lines	Small cell lung cancer	0.792
Saos-2	R158L	Solid tumor cell lines	Osteosarcoma	0.794
U937	R158L	Hematological malignancy cell lines	lymphoma	0.800
SUN668	S215N	Solid tumor cell lines	Stomach Cancer	1.033
SW13	H193Y	Hematological malignancy cell lines	Adrenocortical Cancer	1.061
Detroit562	R175H	Solid tumor cell lines	Pharyngeal cancer	1.070
HEL	M133K	Hematological malignancy cell lines	leukaemia	1.155
Saos-2	V272M	Solid tumor cell lines	Osteosarcoma	1.166
Raji	Y234H	Hematological malignancy cell lines	lymphoma	1.396
SK-BR-3	R175H	Solid tumor cell lines	Breast Cancer	1.41
SHI-1	I195T	Hematological malignancy cell lines	leukaemia	1.700
OVKATE	R282W	Solid tumor cell lines	Ovarian Cancer	1.702
TE-1	V272M	Solid tumor cell lines	Esophageal Cancer	1.823
QG-56	R249S	Solid tumor cell lines	Lung Squamous Cell Carcinoma	1.900
HCT116	R158L	Solid tumor cell lines	Colon Cancer	2.031
HCT116	V272M	Solid tumor cell lines	Colon Cancer	2.128
H1299	V272M	Solid tumor cell lines	Lung Adenocarcinoma	2.198
H1299	R158L	Solid tumor cell lines	Lung Adenocarcinoma	2.822
PATU8988	R282W	Solid tumor cell lines	Pancreatic Cancer	2.886
H441	R158L	Solid tumor cell lines	Lung Adenocarcinoma	3.220
CF	R158L	Solid tumor cell lines	cardiac fibroblasts	3.302
CF	V272M	Solid tumor cell lines	cardiac fibroblasts	3.319
NH91	V143M	Solid tumor cell lines	Lung Squamous Cell Carcinoma	4.329
LK-2	V272M	Solid tumor cell lines	Lung Squamous Cell Carcinoma	5.662

Figure S6

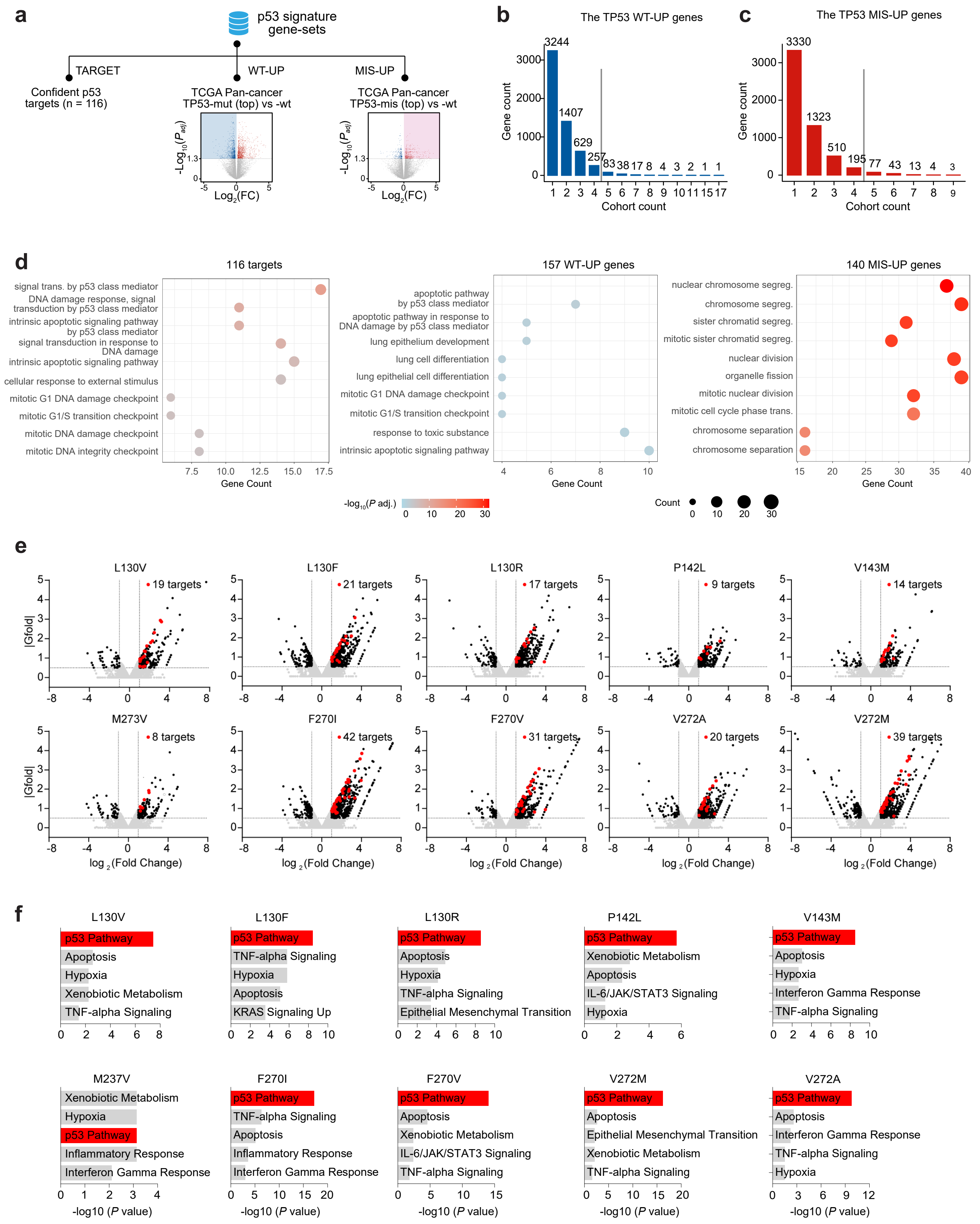

a
p53 signature gene-sets
TARGET
WT-UP
MIS-UP
Confident p53 targets (n = 116)
TCGA Pan-cancer TP53-mut (top) vs -wt
TCGA Pan-cancer TP53-mis (top) vs -wt
b
The TP53 WT-UP genes
Gene count
Cohort count
c
The TP53 MIS-UP genes
d
116 targets
157 WT-UP genes
140 MIS-UP genes
signal trans. by p53 class mediator
DNA damage response, signal transduction by p53 class mediator
intrinsic apoptotic signaling pathway by p53 class mediator
signal transduction in response to DNA damage
intrinsic apoptotic signaling pathway
cellular response to external stimulus
mitotic G1 DNA damage checkpoint
mitotic G1/S transition checkpoint
mitotic DNA damage checkpoint
mitotic DNA integrity checkpoint
apoptotic pathway by p53 class mediator
apoptotic pathway in response to DNA damage by p53 class mediator
lung epithelium development
lung cell differentiation
lung epithelial cell differentiation
response to toxic substance
nuclear chromosome segreg.
chromosome segreg.
sister chromatid segreg.
mitotic sister chromatid segreg.
nuclear division
organelle fission
mitotic nuclear division
mitotic cell cycle phase trans.
chromosome separation
Gene Count
-log10(P adj.)
Count
e
L130V
L130F
L130R
P142L
V143M
M273V
F270I
F270V
V272A
V272M
19 targets
21 targets
17 targets
9 targets
14 targets
8 targets
42 targets
31 targets
20 targets
39 targets
|Gfold|
log 2 (Fold Change)
f
L130V
L130F
L130R
P142L
V143M
M237V
F270I
F270V
V272M
V272A
p53 Pathway
Apoptosis
Hypoxia
Xenobiotic Metabolism
TNF-alpha Signaling
KRAS Signaling Up
Epithelial Mesenchymal Transition
IL-6/JAK/STAT3 Signaling
Interferon Gamma Response
Inflammatory Response
-log10 (P value)
